# Impact of heartfulness meditation practice compared to the gratitude practices on wellbeing and work engagement among healthcare professionals: Randomized trial

**DOI:** 10.1371/journal.pone.0304093

**Published:** 2024-06-07

**Authors:** Kunal Desai, Patricia O’Malley, Emily Van Culin

**Affiliations:** 1 Department of Internal Medicine, Boonshoft School of Medicine, Wright State University, Dayton, Ohio, United States of America; 2 Nurse Scientist- Premier Health Nursing Research, Miami Valley Hospital, Dayton, Ohio, United States of America; 3 Premier Health Learning Institute, Dayton, Ohio, United States of America; Alexandria University Faculty of Nursing, EGYPT

## Abstract

**Objective:**

To investigate whether Heartfulness meditation practice, compared to Gratitude practice, leads to measurable changes in mental wellbeing among healthcare providers across the US.

**Method:**

Participants were randomly assigned to one of the following 6-week interventions: the trainer-guided virtual Heartfulness meditation program or the podcast-based self-guided gratitude practice group. The Professional Quality of Life Scale-5 (ProQOL-5) was used to determine Compassion Satisfaction (CS) and risk for Burnout (BO) and secondary traumatic stress (STS). The Utrecht Work Engagement Scale (UWES) was used to assess vigor, dedication, and absorption at work. Outcomes were collected at baseline and the end of the study period. Qualitative questions regarding the experience of learning and practicing were also offered at the end of the six weeks.

**Results:**

The majority of participants were nurses (50%), followed by allied healthcare professionals (37%) and physicians (13%) (N = 83). There was a general trend towards increases in CS in the Heartfulness group compared to the gratitude group. However, this was not statistically significant. Strong evidence suggests there was a significant improvement in BO for the Heartfulness group between Week 0 and Week 6 (p = 0.002), as well as STS (p = 0.0004) and vigor (p = 0.0392). Qualitative data analysis revealed that the subjects in the Heartfulness arm reported improved sleep and decreased reactivity to stress. Subjects in the gratitude arm reported improved mood and favorable results using gratitude practices at home with family members.

**Conclusion:**

In our study, Heartfulness meditation practice was associated with a significant improvement in burnout and vigor at work, with a trend towards compassion satisfaction after six weeks compared with gratitude practices. Qualitative analysis indicates the benefits of both Heartfulness and Gratitude practices. Further randomized trials with a larger sample size are needed to explore these science-based practices for the wellbeing of healthcare workers.

## Introduction

Stress associated with the work environment is a known phenomenon. When an individual inner coping ability fails to meet high demands, it often results in Stress-related disorders (SRD), including burnout [1]. While involved in the noble profession of helping fellow human beings, healthcare workers (HCWs) are exposed to a challenging work environment where they encounter distressed patients and families. HCWs are significantly affected by SRDs, with increasing prevalence over time [2–4].

Compassion is understood as one’s ability to perceive a fellow being’s distress with a willingness to alleviate it. While compassion and empathy are expected of all healthcare professionals, these constant demands can become overwhelming depending on the individual’s inner coping ability and resilience, different patient populations, and workload. Compassion satisfaction (CS) is generally considered a positive and enriching emotion of satisfaction associated with providing care to patients. Compassion fatigue (CF) is a result of witnessing and absorbing the problems and suffering of others when working with traumatized individuals, leading to emotional and psychological exhaustion [5, 6]. Compassion fatigue is most commonly measured using the *Professional* *Quality of Life* *Scale* (ProQOL), which measures compassion satisfaction (CS), burnout (BO), and secondary trauma stress (STS) [7].

Secondary trauma stress is described as a negative feeling driven by work-related trauma and fear [8]. According to the World Health Organization (WHO), burnout is a syndrome resulting from “chronic workspace stress that has not been successfully managed” and is considered a serious health issue. The result is burnout (BO), evidenced by emotional exhaustion, depersonalization, and a reduced sense of personal accomplishment related to job dissatisfaction [9].

Compassion fatigue and BO affect all disciplines in the healthcare system. Nurses are especially prone to higher CF and BO with unrelenting physical and psychological stress originating from the care of patients, most of whom are seriously ill and many of whom are traumatized [6–10]. Evidence suggests that the prevalence of CF and BO among healthcare workers has increased in recent years [4, 11–13]. A meta-analysis of 21 studies demonstrated that CF, CS, and BO prevalence rates were 47.6%, 52.6%, and 52% among nurses across specialties [13].

The function of the healthcare system depends on the healthy integration and interdependence of the physicians, nurses, and allied healthcare professionals. Allied healthcare workers such as respiratory, physical, occupational, and speech therapists exposed to similar work-related stress while caring for their patients also require CS for practice and wellbeing, even though literature lacks data with regards to these members of the care team [14, 15].

Compassion fatigue and BO are a function of complex factors that include personal and demographic characteristics, healthcare setting, workplace environment, administrative support, shift work, and patient acuity [5, 12]. Moreover, there are serious consequences associated with the increasing prevalence of CF and BO. First, the health of the workforce at an individual level declines with unaddressed CF and BO. Second, associated with CF, BO, and declining health are increased work errors, decreased patient satisfaction, and poorer quality of care [6, 16]. Finally, CF, BO, and declining health are associated with increased turnover, worker’s compensation costs, lawsuits, and, ultimately, a worsening shortage of HCWs, contributing to the institution’s financial strain [6, 17, 18].

Work engagement is a positive, fulfilling, work-related attitude characterized by vigor, dedication, and absorption [19]. and is intrinsically linked with CF and BO [17, 20]. Healthcare workers experiencing high rates of BO and CF are more likely to have lower levels of work engagement and work satisfaction [21].

Health administrators strive to improve the mental wellbeing of their workforce to avoid these consequences. Strategies such as optimal workload assignment, collegial work environment, continuing education, and leadership are critical interventions to help curtail the prevalence of BO and CF. However, depending on one’s inner emotional state, everyone copes differently with stressful work environments or challenging patient or family interactions.

Utilization of self-care tools has been associated with lower CF and BO [21]. There is an increasing need to empower HCWs with self-care tools that are feasible and easily accessible to better cope with day-to-day stressors associated with patient care to enhance resilience and work satisfaction, increase CS, and reduce BO.

Relaxation techniques, mindfulness-based interventions (MBIs), yoga, and meditation practices have been shown to positively impact the wellbeing of HCWs with varied effectiveness [22–24]. Extensive research regarding the effectiveness of MBIs on the wellbeing of HCWs has been conducted, showing an overall positive impact on multiple variables, including stress, anxiety, and burnout [25–29]. In light of the rising CF and BO, there is an urgent need to empower the HCWs with newer approaches and practical self-care tools that can be incorporated into daily living and substantially positively influence wellbeing.

Over the past two decades, research has identified gratitude as significant and observable with favorable effects on health and quality of life. Gratitude is a broad construct in psychology, sociology, and health sciences. As defined by Robert Emmons and Michael McCullough, gratitude is defined as a two-step process: 1. recognition and appreciation of a positive outcome or event and 2. the acknowledgment of the goodness in one’s life and recognizing that much of this goodness was due to forces beyond oneself and through the efforts of others. Gratitude encourages one to “pay it forward,” which enhances happiness and strengthens relationships [30, 31]. Uncovering the role of gratitude for healthcare workers remains an important goal. In a recent meta-narrative review, the authors suggest that gratitude is a significant part of social relations that impacts what persons think, feel, say, or do [32]. Even though gratitude practice could be a promising tool to incorporate into staff education and professional development, current research is lacking in establishing its effectiveness in combating CF and BO among HCWs [33, 34]. Thus, more evidence is necessary to discern the benefits of gratitude practices on wellbeing, job satisfaction, teamwork, absenteeism, and collaboration [32].

Heartfulness Institute is a global nonprofit organization with thousands of volunteer trainers who serve more than a million meditators in over 160 countries (https://www.heartfulnessinstitute.org/). Heartfulness is a simple heart-based meditation practice comprised of relaxation and meditation, rejuvenation techniques, and inner connection to help cultivate poise, resiliency, inner peace, and expansion of consciousness [35, 36]. In recent years, research on Heartfulness meditation practice has shown a positive impact on the wellbeing of different populations, including HCWs. Heartfulness meditation practice effectively decreases burnout and loneliness and improves sleep quality among HCWs [36–38]. Moreover, the virtual accessibility of the Heartfulness program allows for easy accessibility for those who incorporate Heartfulness tools into their current lifestyle [36].

### Study purpose

This study investigated whether a virtual Heartfulness meditation program, compared to gratitude practice, leads to measurable changes in CF, BO, and work engagement (WE) among healthcare providers across the United States. As no study to date examines the effects of Heartfulness meditation practice on CF, CS, and WE among HCWs, any effects and associations uncovered in this research will provide valuable information for now and a basis for future research. Another unique aspect is that this study explores and compares Heartfulness meditation practice with a gratitude practice rather than using a control group for comparison, as in previous studies [37, 38].

## Materials and methods

### Study design

This prospective intervention randomized study was approved by the Wright State University institutional review board (IRB). It was conducted in collaboration with Premier Health Network, Dayton, Ohio, United States of America. The written consent was obtained electronically from each enrolled participant. Any healthcare worker involved in direct patient care at any healthcare facility within the United States was offered an opportunity to participate in the study conducted from October 10, 2022, to November 21, 2022. Anyone with a history of ≥100 hours of meditation practice or anyone person under medical care for depression or other mental health conditions was excluded. Informed consent was obtained from all participants with electronic signatures through an online survey platform approved by IRB. The participants were randomly assigned to one of the two groups (random numbers were assigned for all the participants by the principal investigator with the help of a statistician as follows: after the recruitment was completed, the lowest two-thirds of the random numbers were allocated to the Heartfulness group, while the remaining were allocated to the gratitude group): the Heartfulness meditation group or the Gratitude practice group. Once randomization was done, blinding was not possible due to the types of interventions for both groups. An adaptive recruitment strategy was used with 50% more participants randomly assigned to the meditation practice group (2:1 ratio), which was related to an expected high attrition rate, as reported in the literature [36, 38]. The information regarding the study and invitation to participate was disseminated via email, meetings, and social media platforms such as Facebook using marketing flyers. This study was prospectively registered with the International Standard Randomized Controlled Trial Number (ISRCTN) registry with trial number ISRCTN15778488 (https://doi.org/10.1186/ISRCTN15778488).

### Measures

The Professional Quality of Life Scale-5 (ProQOL-5) was used to determine Compassion Fatigue (CF) risk [7].. ProQOL-5 uses 30 questions discreetly separated into three subscales, assessing Compassion satisfaction (CS), Burnout (BO), and secondary traumatic stress (STS). The reliability of the ProQOL-5 is well documented and has established its reliability with previous research reporting a Cronbach’s α score ranging from 0.71 to 0.8810. Cutoff scores of less than 43 (mild for STS/BO, severe for CS), 43 to 57 (moderate), and 57 or more (severe for STS/BO, mild for CS) were used for analysis. Calculated scores were used to create risk profiles to reflect potential CF trends.

The Utrecht Work Engagement Scale was used in its original version with 17 items [39, 40]. This self-administered instrument consists of seventeen items with Likert-type response scales ranging from 0 (Never) to 6 (Always), distributed over three dimensions: vigor, dedication, and absorption. Vigor refers to the presence of high levels of energy and resilience, willingness to devote efforts, not feeling fatigued easily, and to be persistent in the face of difficulties; dedication refers to the purpose or significance of the work, to feel enthusiastic, proud and inspired by the work done; and absorption refers to feeling so joyful and immersed in work that time passes quickly, and the person forgets what is happening around them. Scores were calculated for each dimension.

All the participants were asked to complete the ProQOL-5 and UWES-17 scale surveys at baseline and at the end of the 6-week study period through the online survey tool Qualtrics.

All participants were invited to provide testimonials or descriptive answers to the qualitative questions in writing as a part of the survey at the end of 6 weeks, and answers were included in the qualitative analysis. The qualitative survey questions are available in the Appendix B in S3 File.

### Intervention

#### Heartfulness meditation group

A virtual orientation session on the aspects of the study and the structure of the meditation protocol was conducted for all the participants in the Heartfulness meditation group. Participants were briefed about expectations during meditation sessions and provided with the trainer’s contact details for any further questions. Weekly virtual live education sessions were conducted regarding the Heartfulness practice during the study period to help the participants understand the importance and nuisances of the practice on various topics, including “*Why meditate*: *Health*, *Happiness*, *and Harmony*,*” Rejuvenate*: *Unburden and gain clarity*,*” “Reconnect for better sleep*,*” Tricks to overcome challenges for new meditators*,*” Tips for deeper meditation experience*,*” Cultivate meditative mind*.*”* Sessions were recorded, and links to video recordings were shared with all study participants to review at their convenience. Audio files were provided with guided relaxation, meditation, and bedtime inner connect.

*Heartfulness virtual trainer-guided sessions*. Ten virtual guided relaxation and meditation sessions per week lasting 20–25 minutes during morning times and seven guided rejuvenation sessions in the evenings per week were conducted by one of the authors and a certified Heartfulness trainer (KD) seven days a week for the 6-week study period. Participants were advised to attend at least two guided sessions- one each of meditation and rejuvenation per week. Heartfulness relaxation, meditation, rejuvenation, and bedtime inner connect techniques are provided in the Appendix C in S3 File and are consistent with the interventions described in the previous studies [36, 37].

*Heartfulness self-practice*. Participants were suggested the following Heartfulness techniques to practice to the best of their abilities.

1. In the morning, the participants were to listen to an audio file consisting of the Heartfulness relaxation technique followed by meditation for about 15–30 minutes in place of or in addition to guided sessions as per the participants’ convenience.
2. In the evening or at the end of the day’s work, the participants were to listen to an audio file consisting of the Heartfulness relaxation technique followed by rejuvenation for about 15–20 minutes in place of or in addition to guided sessions as per the convenience of the participants.
3. Participants were to listen to an audio file of the Heartfulness Inner Connect at night, followed by relaxation. This was to be completed just before going to sleep.

#### Gratitude practice group

Recent research has brought forth many gratitude practices for implementation and research. For this study, the gratitude arm provided a weekly podcast with a short newsletter document via email for subjects. Each podcast was 50 minutes long and could be accessed anytime after the link was sent to the subject via email each Tuesday for five weeks. The investigators provided and role-played two or three gratitude practices during the discussion in each of the five podcasts. Sources for podcast discussions and practices were: 1. Gratitude Practice for Nurses: A Toolkit for Wellbeing by the American Nurses Foundation (ANF) and Greater Good Science Center (GGSC), (Available at: https://ggsc.berkeley.edu/images/uploads/Gratitude_Nurses_Toolkit.pdf, Accessed July 27, 2023), Gratitude Practice for Nurses- Implementation Guide to the Toolkit for Wellbeing. (Available at: https://www.nursingworld.org/~4a22d0/globalassets/covid19/implementation_guide.pdf, Accessed July 27, 2023, and 3. Greater Good Toolkit- 30 Science-Based Practices for a Meaningful Life. Holstee, Brooklyn, New York. 2020. (Available at: https://www.holstee.com, Accessed July 27, 2023).

Each of the five weekly gratitude podcasts explored gratitude practices and linked each practice with the *Greater Good in Action* website https://ggia.berkeley.edu/practice/ so that the subject could further explore the gratitude practice after the podcast. Subjects were asked to try to practice one or more of the gratitude tools explored in the podcast for the week and review the newsletter received via email that summarized the gratitude practice and provided additional references and resources. A table provided in the Appendix A in S3 File describes the podcast schedule and content for the 5-week gratitude arm of the study. Sixteen of the possible 30 gratitude practices described by Greater Good Science Center were explored with participants during the study. All tools are available at Greater Good in Action (berkeley.edu).

All participants were asked to self-report their adherence to the intervention at the end of the 6-week study period. A research team tracked attendance in virtual heartfulness and gratitude sessions.

### Statistical analysis

We assessed the demographic characteristics for both groups using Fisher’s exact test. We analyzed each of the six sub-scales of ProQOL-5 and The Utrecht Work Engagement Scale using repeated measures ANOVA. The independent variable was a group (Heartfulness or Gratitude Practice), and the repeated measure was time (0 weeks and six weeks). Participants were considered adherent to the program if they practiced meditation and/or rejuvenation on average at least four times a week. Thus, participants were only included in the between-group analysis if their mean participation per week value (including self-practice) was at least four. All the participants from the Gratitude practice who filled out the Week 6 survey indicated engaging in at least some gratitude practice activities, so none of their Week 6 data was excluded from the analysis. The analysis was completed per the study protocol. Intention to treat analysis is not performed.

The full model, which included the interaction between time and group, was run since it was hypothesized that changes in scores across time would not be the same for both groups. Comparisons of interest include between the two groups at 0 weeks and six weeks and between 0 and 6 weeks within each group. Post-hoc multiple comparisons were made via the sequentially rejective Bonferroni procedure [41].. This procedure adjusts the p-values to account for a potentially inflated type I error rate (false positive rate) that can occur when multiple comparisons are made and ensures that the overall type I error rate of each repeated measures ANOVA is at most α = 0.05. All statistical tests were two-tailed.

A level of significance of α = 0.05 will be used throughout to assess statistical significance, and SAS version 9.4 (SAS Institute, Inc., Cary, NC) was used for all analyses.

## Results

### Participant characteristics

**Fig 1:**
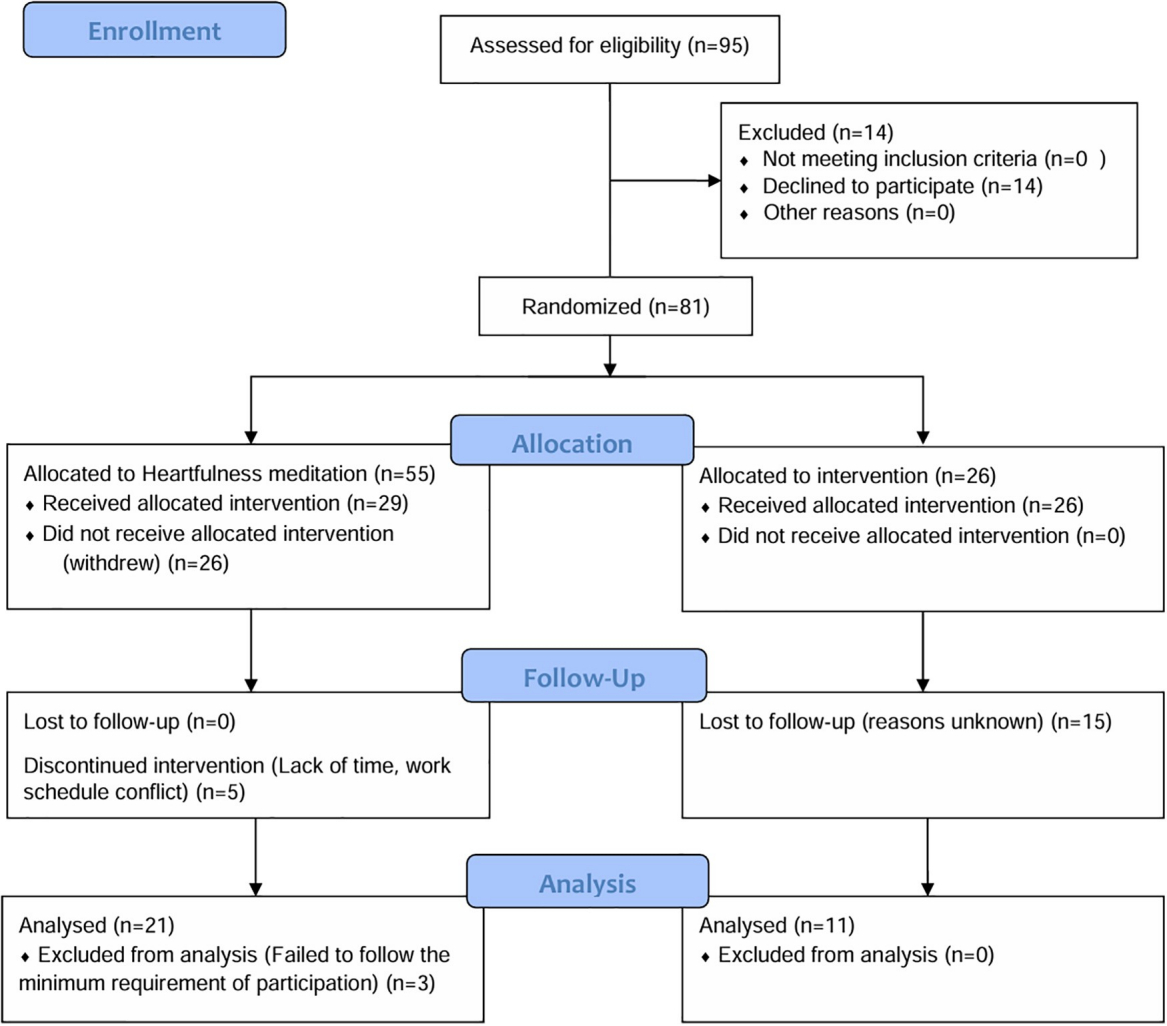
Participant flowchart based on the CONSORT guidelines.

Ninety-five healthcare workers expressed interest in participating, and fourteen declined to participate. The remaining 81 were randomized to the Heartfulness meditation group (n = 55) or the Gratitude practice group (n = 26) (Fig 1). A total of eighty-one participants completed the baseline survey. Participants’ characteristics are summarized in Table 1. There were no baseline differences between groups in sociodemographic variables. The mean age was 45 years (SD = 11.96). There were 71 females (85.6%) and ten males (12.3%). Most of the participants were nurses (50%), followed by allied healthcare professionals (37%) and physicians (13%).

**Table 1:** Participants baseline demographic characteristics by groups (N = 81).

Baseline characteristic	Heartfulness Group (n = 55)		Gratitude Group (n = 26)		P value
	*n*	%	*n*	%	
**Gender**					
Female	51	92.7	20	76.9	0.07*
Male	4	7.3	6	23.1	
					
**Work Designation**					
Nursing	33	60	8	30.8	0.0246*
Allied healthcare professional	18	32.7	12	46.2	
Physician	4	7.3	6	23.1	
					
**How many years of experience do you have in the current profession or position?**					
1–9 years	22	40	10	40	0.74*
10–19 years	11	20	8	32	
20–29 years	8	14.6	3	12	
30–39 years	12	21.8	3	12	
More than 40 years	2	3.6	1	4	
					
**Level of education**					
Associate degree	4	7.3	4	15.4	0.0374*
Bachelor’s degree	19	34.6	9	34.6	
Doctorate degree	5	9.1	5	19.2	
Master’s degree	24	43.6	4	15.4	
Professional Degree	2	3.6	4	15.4	
Some college credit, no degree	1	1.8	0	0	
					
**Have you worked in COVID-19-designated nursing units or High-risk respiratory units (HRRU) in the last year?**					
Yes	19	34.55	7	26.9	0.49*
No	36	65.45	19	73.1	
					
**What is your typical work schedule?**					
Day shifts	37	68.5	20	76.9	0.50*
Night Shifts	9	16.7	1	3.9	
Random or unpredictable	4	7.4	2	7.7	
Some days and some nights	2	3.7	2	7.7	
Other	2	3.7	1	3.9	

* Fisher’s exact test

#### Effects on CS, BO, and STS

**Fig 2:**
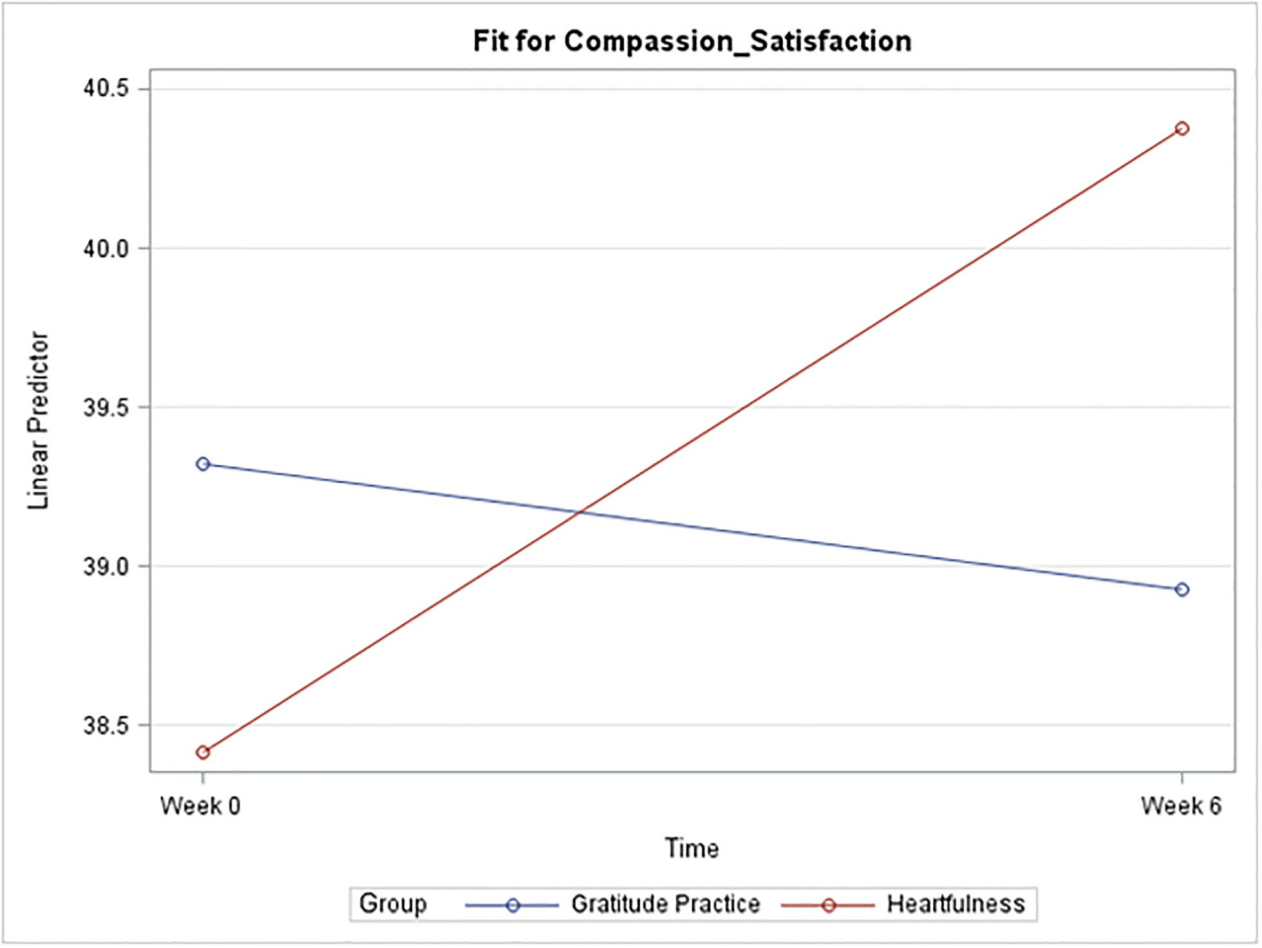
Plot of mean compassion satisfaction by group and time.

Descriptive statistics for CS, BO, and STS broken down by time and group are described in Table 2 and Fig 2, followed by the results of the sequentially rejective Bonferroni procedure in Table 3.

**Fig 3:**
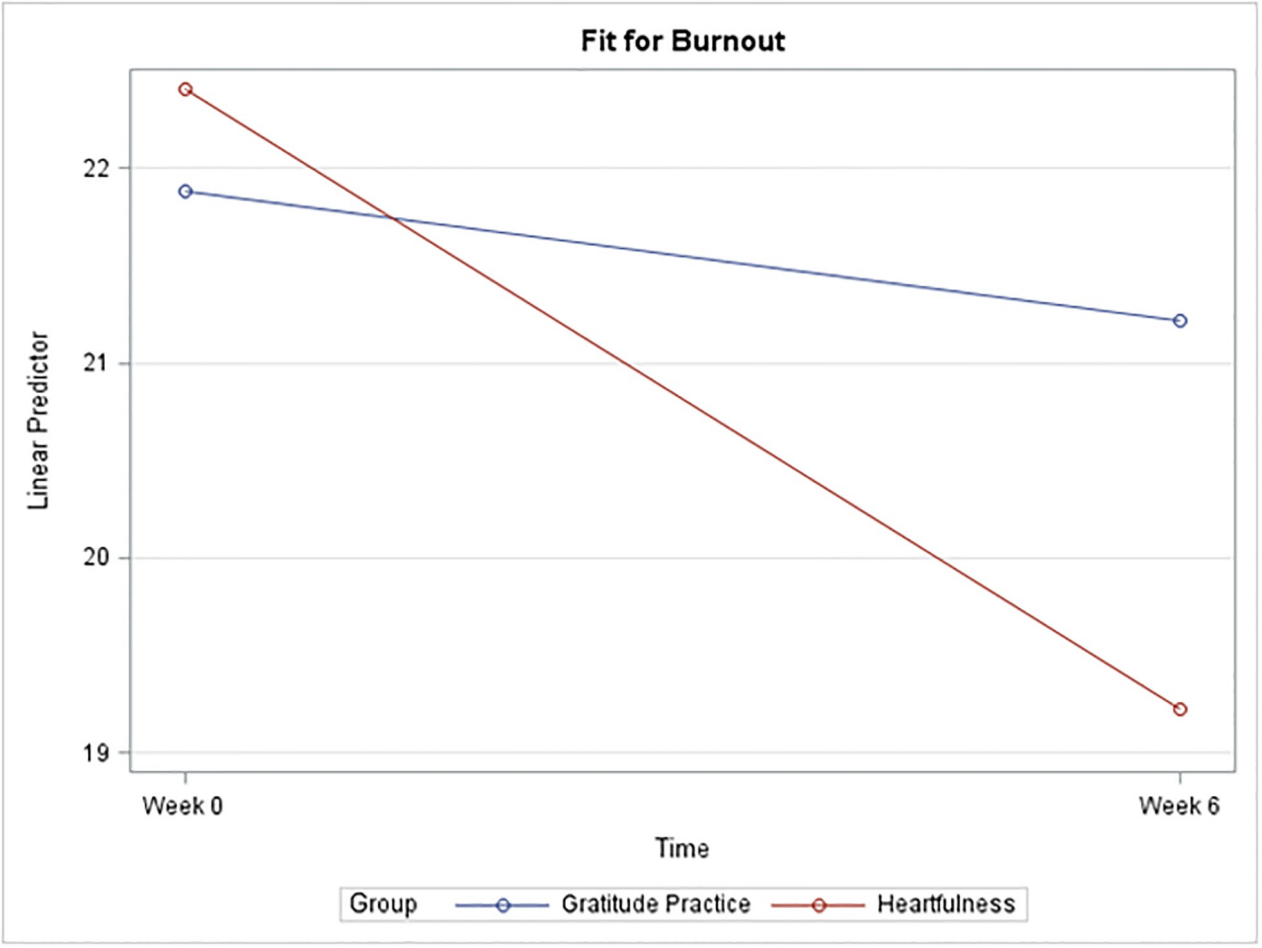
Plot of mean Burnout by group and time.

**Fig 4:**
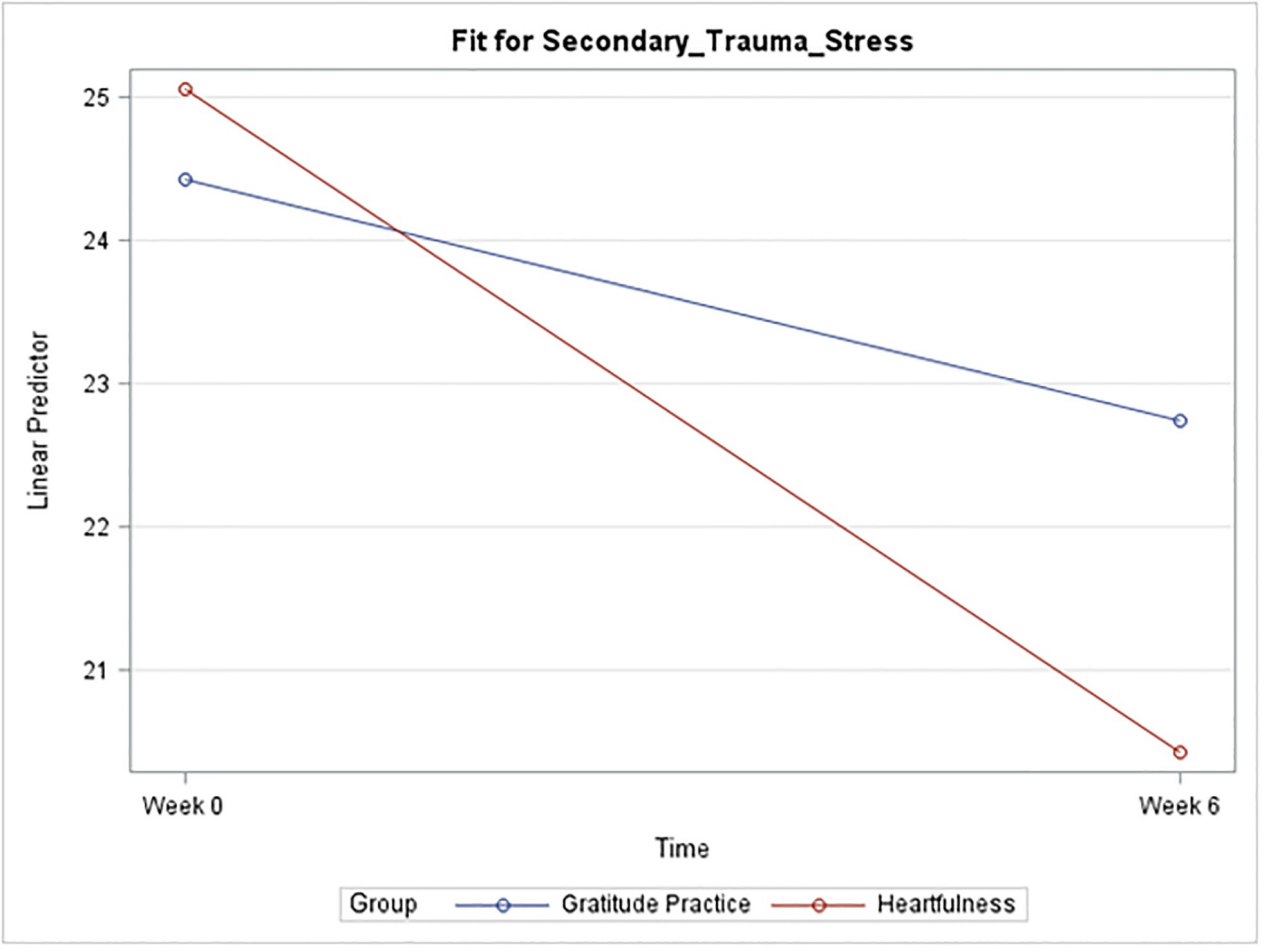
Plot of mean secondary trauma stress by group and time.

*I*. *Compassion satisfaction*. A plot of the mean CS score for each group at each time is given below in Fig 1. A general trend towards increases in CS with the Heartfulness group compared to the gratitude practice group was observed, but it was not enough to be statistically significant (Fig 2).
*II*. *Burnout*. Strong evidence suggests a significant mean difference in burnout score for the Heartfulness group between Week 0 and Week 6 (p = 0.002). The estimated mean difference is 3.35 points lower at Week 6. A 95% confidence interval (C.I.) for the true mean difference among all such participants is (-5.61, -1.1). Strong evidence also suggests a significant mean difference between the two groups at Week 6 (p = 0.0074). The estimated mean difference is 4.42 points lower for the Heartfulness group (95% CI (-8.03, -0.82)) [Fig 3].
*III*. *Secondary trauma stress*. Strong evidence suggests a significant mean difference in the STS score for the Heartfulness group between Week 0 and Week 6 (p = 0.0004). The estimated mean difference is 5.39 points lower at Week 6 (95% C.I. (-8.54, -2.25)). Strong evidence also suggests a significant mean difference between the two groups at Week 6 (p = 0.0356). The estimated mean difference is 4.71 points lower for the Heartfulness group (95% CI (-9.38, -0.04)). [Fig 4].

**Table 2:** Descriptive statistics for CS, BO, and STS by group and time.

		CS		BO		STS	
Group	Time	Mean	Standard Error	Mean	Standard Error	Mean	Standard Error
Gratitude Practice	Week 0	39.32	0.99	21.91	0.75	24.47	0.94
	Week 6	38.91	1.36	21.01	1.05	22.73	1.39
Heartfulness	Week 0	39.93	1.25	19.94	0.93	23.41	1.14
	Week 6	42.47	1.23	16.59	0.93	18.02	1.17

**Table 3:** Results of sequentially rejective Bonferroni procedure for CS, BO, and STS.

	CS			BO			STS		
	Mean Difference	p-value	95% C.I.	Mean Difference	p-value	95% C.I.	Mean Difference	p-value	95% C.I.
Gratitude Practice: Week 6 vs Baseline	-0.41	0.99	(-3.82, 3)	-0.90	0.3800	(-3.58, 1.78)	-1.74	0.4600	(-5.48, 2.01)
Heartfulness: Week 6 vs Baseline	2.54	0.11	(-0.37, 5.46)	**-3.35**	**0.0020**	**(-5.61, -1.1)**	**-5.39**	**0.0004**	**(-8.54, -2.25)**
Week 0: Heartfulness vs Gratitude Practice	0.60	0.99	(-3.51, 4.72)	-1.97	0.2100	(-5.07, 1.13)	-1.05	0.4800	(-4.86, 2.75)
Week 6: Heartfulness vs Gratitude Practice	3.56	0.17	(-1.15, 8.26)	**-4.42**	**0.0074**	**(-8.03, -0.82)**	**-4.71**	**0.0356**	**(-9.38, -0.04)**

#### Effects on work engagement

Descriptive statistics for vigor, dedication, and absorption broken down by time and group are given in Table 4, followed by the results of the sequentially rejective Bonferroni procedure in Table 5

**Table 4:** Descriptive statistics for work engagement by group and time.

		Vigor		Dedication		Absorption	
Group	Time	Mean	Standard Error	Mean	Standard Error	Mean	Standard Error
Gratitude Practice	Week 0	4.27	0.19	4.71	0.19	4.08	0.24
	Week 6	4.35	0.25	4.81	0.26	4.10	0.31
Heartfulness	Week 0	4.46	0.23	4.66	0.23	3.87	0.29
	Week 6	5.02	0.23	5.14	0.23	3.87	0.29

**Table 5:** Results of sequentially rejective Bonferroni procedure for work Engagement.

	Vigor			Dedication			Absorption		
	Mean Difference	p-value	95% C.I.	Mean Difference	p-value	95% C.I.	Mean Difference	p-value	95% C.I.
Gratitude Practice: Week 6 vs Baseline	0.08	0.9900	(-0.57, 0.74)	0.10	0.99	(-0.57, 0.76)	0.03	0.99	(-0.72, 0.77)
Heartfulness: Week 6 vs Baseline	**0.56**	**0.0392**	**(0.02, 1.1)**	0.48	0.10	(-0.06, 1.02)	0.00	0.99	(-0.57, 0.57)
Week 0: Heartfulness vs Gratitude Practice	0.19	0.9900	(-0.57, 0.95)	-0.05	0.99	(-0.82, 0.71)	-0.20	0.99	(-1.18, 0.78)
Week 6: Heartfulness vs Gratitude Practice	0.67	0.1600	(-0.21, 1.54)	0.34	0.99	(-0.55, 1.22)	-0.23	0.99	(-1.32, 0.86)

**Fig 5:**
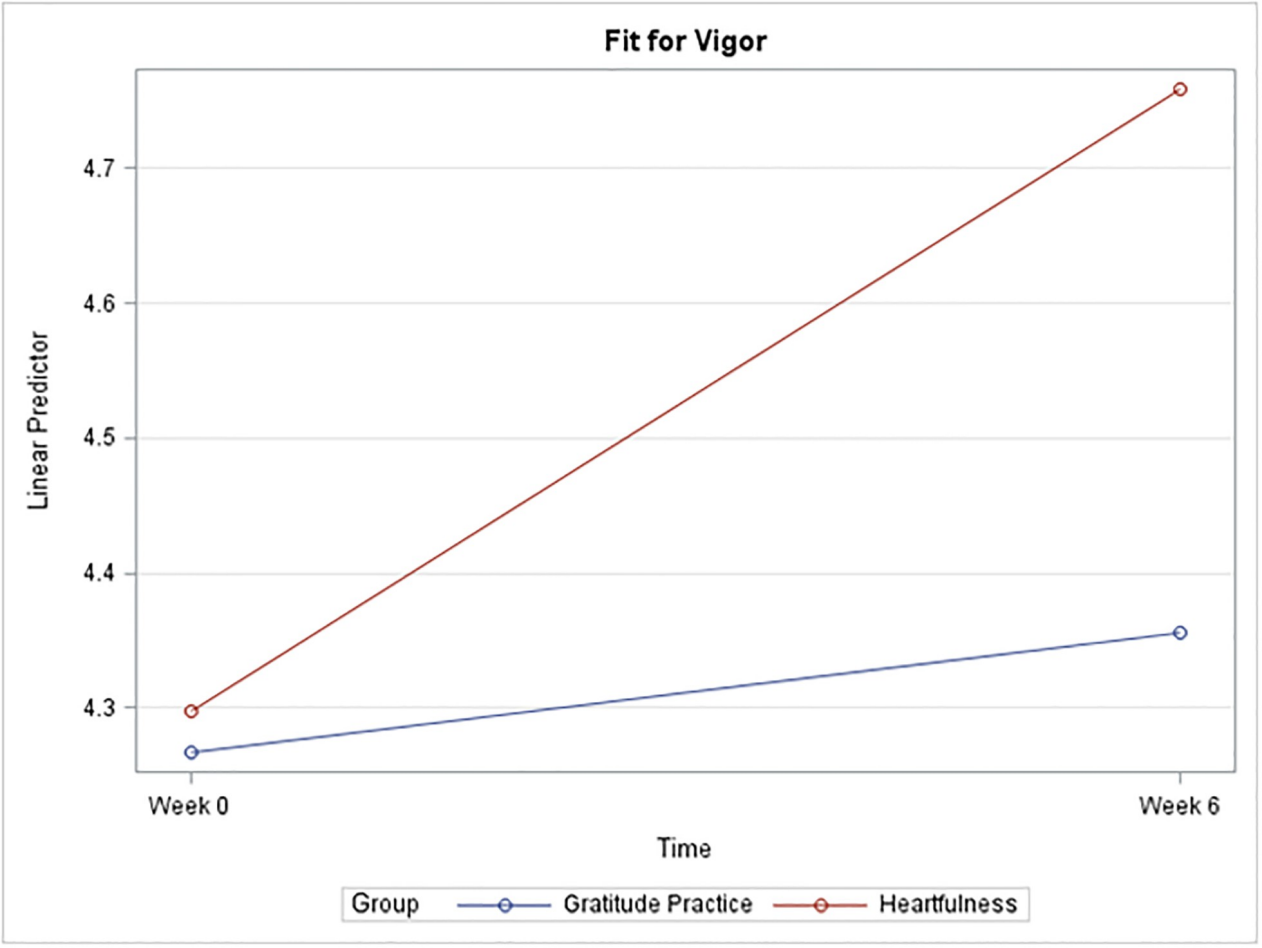
Plot of mean vigor by group and time.

Strong evidence suggests a significant mean difference in the vigor score for the Heartfulness group between Week 0 and Week 6 (p = 0.0392). The estimated mean difference is 0.56 points higher at Week 6 (95% C.I. (0.02, 1.1)) [Fig 5]. There was insufficient evidence to suggest any difference in dedication and absorption between the two groups.

#### Adherence to practice

Among the participants in the Heartfulness group, 48.3% and 10.3% of participants who completed the 6-week survey engaged in at least two Heartfulness meditation sessions on their own per week and attended at least two guided meditation sessions per week, respectively. 48.3% and 14% of participants who completed the 6-week survey engaged in at least two rejuvenation sessions on their own per week, and 14% attended at least two guided rejuvenation sessions per week, respectively.

### Qualitative analysis

Participants who completed the study were provided the opportunity to respond to an electronic survey with open-ended questions regarding the impact of participation on wellbeing, relationships, and professional life. Participants could also describe the challenges of the study and offer ideas on how the study could be improved and the benefits obtained with participation.

Thirty-eight participants Heartfulness group n = 27, and gratitude group, n = 11) provided feedback. Results are described in Appendix D in S2 File. *Subject Comments Heartfulness Training* and Appendix E in S1 File. *Subject Comments Gratitude Practices*.

There were many similar benefits for Heartfulness and gratitude groups. Both groups report reduced stress, feelings of calm, feeling less reactive, and a deeper appreciation of self, family, and others. Additionally, both groups reported increased empathy and patience. Finally, both groups did report the challenge of finding time to attend virtual Heartfulness training consistently or to listen to gratitude podcasts.

There were differences in self-reports for the two groups. The Heartfulness group reported improved sleep and less fatigue, which was not reported in the gratitude group. The gratitude group reported sharing gratitude practices with family members and modifying time management to take more time for self and family, which was not reported in the Heartfulness cohort.

The benefits of Heartfulness training included increased compassion and learning to create balance and manage stress, and the instructor-led sessions helped keep one accountable. As for gratitude, participants reported practices helped with “recalibrating” and would be used in the future for difficult moments. Both groups shared the desire to hear about others’ experiences with Heartfulness and gratitude. Finally, while podcasts were reported as effective, participants requested more visual or live communication in future research.

## Discussion

Meditation practices have been shown to be beneficial as an intervention to address CF in the nursing population. However, some of these studies had small sample sizes and lacked a control group [42, 43]. Loving-kindness meditation significantly reduced CF in the nurses working in neonatal intensive care after one month in an RCT [44]. To the best of our knowledge, ours is the first study that has assessed the effect of Heartfulness meditation practice compared to gratitude practice on CF, BO, and work engagement. Our results show that the intervention of a trainer-guided virtual Heartfulness meditation program teaching self-care practice of simple heart-based meditation, rejuvenation, and inner connect was associated with a statistically significant reduction in BO (17%) and STS (23%) with a trend towards an increase in CS (6.5%) among the nurses and allied healthcare professionals after six weeks when compared to self-guided podcasts for gratitude practices.

Moreover, when work engagement was assessed within this cohort, there was a statistical increase in vigor (12.5%) and a trend toward an increase in dedication (10%) with no change in absorption at six weeks among the Heartfulness group compared to the gratitude practice group. Our findings further strengthen the potential of the Heartfulness meditation practice in improving the psychological wellbeing observed in the previous studies [36–38, 45–47], even when compared with another wellness intervention instead of a control group.

A recent study also reported a significant reduction in cortisol levels and an increase in telomere length in healthy volunteers after 12 weeks of Heartfulness meditation. This study that provides evidence of biological parameters associated with stress-related disorders supports use as a possible outcome measure for Heartfulness practices in future research [45]. In other recent studies, a mindfulness online course was associated with improved work engagement, though the study lacked a control group [48]. and the benefits of mindfulness on work engagement among non-healthcare subjects compared to waitlist control [49]. This study is the first to assess CF, BO, and work engagement among HCWs practicing Heartfulness versus gratitude practices.

### Gratitude science

A multi-year project began between the Greater Good Science Center and Robert Emmons of the University of California Davis, identified as *Expanding the Science and Practice of Gratitude*, and was funded by the John Templeton Foundation in 2014. Researchers across the United States received nearly $4 million in funding to explore the transforming benefits of gratitude on the brain as a key to building resilience, a meaningful life, and a stronger community. Study findings revealed that those who practice gratitude report fewer symptoms of illness and depression, have greater optimism and happiness, and have stronger, more positive relationships [31]. Research is lacking in assessing the impact of the gratitude practice in addressing burnout and CF among HCWs [50–52]. In a non-randomized trial, a simple daily gratitude journaling intervention called “three good things” was compared to a control group among Pediatric resident physicians, which showed a 10% decrease in one measure of burnout. However, overall, the results of most outcomes in this study were nonsignificant, and the sample size was small [51]. A study assessed the effects of a gratitude journal for reducing burnout in healthcare workers at an elderly care facility in the US and found that for the 25 participants of the study who completed the pre-and post-intervention measures, there was no significant improvement in burnout over the 21 days in which participants were asked to keep a daily gratitude journal. However, participants expressed appreciation and enjoyment of completing the journal [50]. Our study showed no significant change in measured variables in the gratitude practice group. However, the qualitative analysis demonstrated the positive influence of the gratitude practice, where the participants reported feelings of calm, feeling less reactive, and improved relationships reflected by spending time and sharing gratitude with family.

### Heartfulness science & mindfulness

A systemic review of 11 randomized control trials (RCTs) conducted between 2011 and 2021 assessing the effectiveness of MBIs on the psychological wellbeing of nurses demonstrated that MBIs effectively reduced stress and burnout in nurses [29]. Another meta-analysis of 14 studies, including 1077 nurses, also noted a similar impact of MBIs [25]. However, there was variability in the length of mindfulness practices in different studies, and evidence of dose-response effects was mixed and concluded not to support strong causal conclusions. A meta-analysis of 10 studies between 2009 and 2020 testing the effects of MBIs on BO among healthcare professionals found that the studies had a high risk of bias and limited quality evidence but enough to recommend the implementation of MBIs to address burnout [53].

#### Proposed mechanism of action

Heartfulness practice offers an individual an intentional pause through meditation, resulting in a feeling of calmness and inner connection. The repeated experience of this calmness slowly percolates in one’s day-to-day activities and helps cope with difficult circumstances with poise and balanced emotional response. In addition, the Heartfulness rejuvenation practice recommended at the end of the day helps remove the emotional burden. Previous work has also proposed the cultivation of resiliency and inner peace [36]. Moreover, it becomes natural for one to express compassion when there is a feeling of inner contentment and calmness. These are potential mechanisms for how this intervention can help reduce burnout and improve compassion satisfaction.

### Going forward: The conceptual differences in mindfulness, heartfulness, and gratitude sciences

While mindfulness interventions have been extensively studied, which often require variable amounts of time for training with varied effectiveness, the gratitude practice seems to take less time to learn and implement in daily routine. However, gratitude practices lack robust research data to support their effectiveness. It, therefore, might be imperative to look for impactful, practical, and feasible interventions to apply in the busy and challenging work schedules of HCWs.

There is one obvious similarity between the MBIs, gratitude practice, and the Heartfulness meditation practice, which encourages individuals to take some time to pause, turn inward, and get in touch with their emotions and feelings to some extent. Gratitude practice promotes consciously cultivating feelings of contentment and gratefulness to others. Mindfulness involves the practice of meditation with relatively more active awareness, exploration, and nonjudgmental understanding of cognitions and emotions [54].

Heartfulness practice involves meditation with a simple intention-setting only at the start, to suppose the presence of the light within the heart, also referred to as the Source or “inner Self,” while encouraging to keep the passive attitude, designated to help regulate and relax the mind eventually leading to cultivate meditative mind [35]. The use of yogic transmission is described as the most distinguishing feature of Heartfulness, allowing the practitioner to take their awareness to deeper levels or dimensions of Self [35]. Heartfulness practice is proposed to cultivate emotions including peace, serenity, love, acceptance, humility, service, compassion, and empathy [55]. The participants who completed eight weeks of Heartfulness practice were able to cultivate a quality of empathy, acceptance, and individual peace according to the qualitative thematic analysis [36]. Considering these subtle but essential conceptual differences between these techniques, we propose that the Heartfulness meditation practice might effectively combat burnout and promote higher compassion satisfaction among HCWs.

Moreover, the qualitative analysis of the current study demonstrated that both Heartfulness and gratitude practice interventions appear to positively impact wellbeing, relationships, and professional life. Participants reported being highly/extremely likely to recommend these two wellness interventions to family or friends. Our findings suggest benefits and opportunities for future research on both Heartfulness and gratitude practices.

### Limitations

Despite the utility of our findings, the study contains several limitations. The sample size was small and attributed to high attrition rates in both the Heartfulness and gratitude practice groups. The attrition rate was high but similar to the observation in previous studies with the application of a virtual platform for the intervention [35, 56]. Perceived lack of time commitment was reported as one of the common reasons for dropping out.

We speculate that other factors, such as stress related to work, home situations, changes in work schedule, virtual mode of intervention in the Heartfulness group, and lack of human connection with podcast-driven intervention in the gratitude group, might have contributed to the attrition. Certain measured parameters did not show a significant change after the practices of Heartfulness or gratitude. One reason could be because of a small sample size, and the second could be the intervention duration of only six weeks. Another factor that might have influenced the results could be different intervention delivery methods in both groups.

Despite both wellness interventions utilizing the virtual platform, the participants in the Heartfulness group could interact with others and a Heartfulness trainer during the group education or meditation sessions. In contrast, the participants in the gratitude practice group might have required more self-motivation to follow the program.

### Recommendations for future research

The high rates of BO and CF remain significant concerns for healthcare organizations. Evidence suggests that Heartfulness meditation is one of the potentially promising options to address unfavorable consequences of stress, BO, and CF among HCWs at individual and organizational levels. Future studies need to explore this further with a larger sample size, longer duration of intervention, and assessment of sustained effects. Efforts should be directed to reduce attrition by looking into hybrid methods of intervention, both in-person and virtual, as well as assignment of personal Heartfulness trainers to study participants (Heartfulness trainers are meditators and volunteers who have taken up the duty of training others in the practices of Heartfulness, source: www.heartfulness.org).

Regarding gratitude practice, more human interactions can be considered in addition to podcast-based education. Moreover, wellness and self-care tools can be studied while making them integral to the nursing, medical students, and allied healthcare profession education curriculum.

We also suggest a longer follow-up period of 6 to 12 months in future studies to explore and assess the sustainability of the interventions’ effectiveness. Moreover, there might be merit in studying the results of combining Heartfulness and gratitude practices as they could potentially complement each other.

## Conclusion

In our study, Heartfulness meditation practice was associated with a significant improvement in BO and vigor at work, with a trend towards CS after six weeks compared with gratitude practices. Qualitative analysis indicates the benefits of both Heartfulness and gratitude practices. Further randomized trials with a larger sample size are needed to explore these science-based practices for the sustainable impact on the wellbeing of healthcare workers. We also propose healthcare administration-driven efforts to promote such research endeavors at a larger scale in the future.

## Supporting information

S1 Checklist CONSORT 2010 checklist of information to include when reporting a randomised trial*. (DOC)

S2 Checklist Human participants research checklist. (DOCX)

S1 File (DOCX)

S2 File (DOCX)

S3 File (DOCX)

S1 Protocol (PDF)

## References

[1] Nieuwenhuijsen K, Bruinvels D, Frings-Dresen M. Psychosocial work environment and stress-related disorders, a systematic review. Occup Med (Chic Ill) [Internet]. 2010 [cited 2023 Nov 16];60:277–86. Available from: https://academic.oup.com/occmed/article/60/4/277/1392515 doi: 10.1093/occmed/kqq081 20511268

[2] Emal LM, Tamminga SJ, Daams JG, Kezic S, Timmermans DRM, Schaafsma FG, et al. Risk communication about work-related stress disorders in healthcare workers: a scoping review. Int Arch Occup Environ Health [Internet]. 2022 Aug 1 [cited 2023 Nov 16];95(6):1195–208. Available from: https://link.springer.com/article/10.1007/s00420-022-01851-x 35292839 10.1007/s00420-022-01851-x PMC8923828

[3] Ruiz-Fernández MD, Ramos-Pichardo JD, Ibáñez-Masero O, Cabrera-Troya J, Carmona-Rega MI, Ortega-Galán ÁM. Compassion fatigue, burnout, compassion satisfaction and perceived stress in healthcare professionals during the COVID-19 health crisis in Spain. J Clin Nurs [Internet]. 2020 Nov 1 [cited 2021 Aug 20];29(21–22):4321–30. Available from: https://pubmed-ncbi-nlm-nih-gov.ezproxy.libraries.wright.edu/32860287/ 32860287 10.1111/jocn.15469

[4] Lluch-Sanz C, Galiana L, Doménech-Vañó P, Sansó N. The Impact of the COVID-19 Pandemic on Burnout, Compassion Fatigue, and Compassion Satisfaction in Healthcare Personnel: A Systematic Review of the Literature Published during the First Year of the Pandemic. Healthcare (Switzerland). 2022 Feb 1;10(2). 10.3390/healthcare10020364 PMC8872521 35206978

[5] Hunsaker S, Chen HC, Maughan D, Heaston S. Factors That Influence the Development of Compassion Fatigue, Burnout, and Compassion Satisfaction in Emergency Department Nurses. Journal of Nursing Scholarship [Internet]. 2015 Mar 1 [cited 2023 Nov 16];47(2):186–94. Available from: https://onlinelibrary.wiley.com/doi/full/10.1111/jnu.12122 25644276 10.1111/jnu.12122

[6] Peters E. Compassion fatigue in nursing: A concept analysis. Nurs Forum (Auckl). 2018;53(4):466–80. 10.1111/nuf.12274 29962010

[7] Stamm BH. The Concise ProQOL Manual. 2010;

[8] Hooper C, Craig J, Janvrin DR, Wetsel MA, Reimels E. Compassion Satisfaction, Burnout, and Compassion Fatigue Among Emergency Nurses Compared With Nurses in Other Selected Inpatient Specialties. J Emerg Nurs. 2010 Sep 1;36(5):420–7. doi: 10.1016/j.jen.2009.11.027 20837210

[9] Maslach Burnout Inventory: Third edition. [Internet]. [cited 2023 Nov 16]. https://psycnet.apa.org/record/1997-09146-011

[10] Zhang YY, Han WL, Qin W, Yin HX, Zhang CF, Kong C, et al. Extent of compassion satisfaction, compassion fatigue and burnout in nursing: A meta-analysis. J Nurs Manag. 2018;26(7):810–9. doi: 10.1111/jonm.12589 30129106

[11] Cavanagh N, Cockett G, Heinrich C, Doig L, Fiest K, Guichon JR, et al. Compassion fatigue in healthcare providers: A systematic review and meta-analysis. Nurs Ethics [Internet]. 2020 May 1 [cited 2023 Nov 16];27(3):639–65. Available from: https://journals.sagepub.com/doi/abs/10.1177/0969733019889400?journalCode=neja 31829113 10.1177/0969733019889400

[12] Ruiz-Fernández MD, Pérez-García E, Ortega-Galán ÁM. Quality of life in nursing professionals: Burnout, fatigue, and compassion satisfaction. Int J Environ Res Public Health. 2020 Feb 2;17(4). 10.3390/ijerph17041253 PMC7068555 32075252

[13] Xie W, Chen L, Feng F, Okoli CTC, Tang P, Zeng L, et al. The prevalence of compassion satisfaction and compassion fatigue among nurses: A systematic review and meta-analysis. Int J Nurs Stud [Internet]. 2021 [cited 2023 Nov 16];120:103973. Available from: doi: 10.1016/j.ijnurstu.2021.103973 34102372

[14] Sorenson C, Bolick B, Wright K, Hamilton R. Understanding Compassion Fatigue in Healthcare Providers: A Review of Current Literature. Journal of Nursing Scholarship. 2016;48(5):456–65. doi: 10.1111/jnu.12229 27351469

[15] Smart D, English A, James J, Wilson M, Daratha KB, Childers B, et al. Compassion fatigue and satisfaction: A cross-sectional survey among US healthcare workers. Nurs Health Sci. 2014;16(1):3–10. doi: 10.1111/nhs.12068 23663318

[16] Panagioti M, Geraghty K, Johnson J, Zhou A, Panagopoulou E, Chew-Graham C, et al. Association Between Physician Burnout and Patient Safety, Professionalism, and Patient Satisfaction: A Systematic Review and Meta-analysis. JAMA Intern Med [Internet]. 2018 Oct 1 [cited 2023 Nov 16];178(10):1317–31. Available from: https://jamanetwork.com/journals/jamainternalmedicine/fullarticle/2698144 30193239 10.1001/jamainternmed.2018.3713 PMC6233757

[17] Cao X, Chen L. Relationships between resilience, empathy, compassion fatigue, work engagement and turnover intention in haemodialysis nurses: A cross-sectional study. J Nurs Manag [Inter0net]. 2021 Jul 1 [cited 2023 Nov 16];29(5):1054–63. Available from: https://onlinelibrary-wiley-com.ezproxy.libraries.wright.edu/doi/full/10.1111/jonm.13243 33393134 10.1111/jonm.13243

[18] De Simone S, Planta A, Cicotto G. The role of job satisfaction, work engagement, self-efficacy and agentic capacities on nurses’ turnover intention and patient satisfaction. Appl Nurs Res [Internet]. 2018 Feb 1 [cited 2023 Nov 16];39:130–40. Available from: https://pubmed.ncbi.nlm.nih.gov/29422148/29422148 10.1016/j.apnr.2017.11.004

[19] Schaufeli WB, Salanova M, Bakker AB, Gonzales-Roma V. The Measurement of Engagement and Burnout: A two sample confirmatory Factor Analytic Approach. J Happiness Stud [Internet]. 2002 [cited 2023 Nov 16];3(1):71–92. Available from: https://link-springer-com.ezproxy.libraries.wright.edu/article/10.1023/A:1015630930326

[20] Gómez-Salgado Rn J, Domínguez-Salas S, Romero-Martín M, Romero A, Coronado-Vázquez Valle |, Ruiz-Frutos C, et al. Work engagement and psychological distress of health professionals during the COVID-19 pandemic. J Nurs Manag [Internet]. 2021 Jul 1 [cited 2022 Jul 9];29(5):1016–25. Available from: https://onlinelibrary.wiley.com/doi/full/10.1111/jonm.13239 33400325 10.1111/jonm.13239

[21] Cuartero-Castañer ME, Hidalgo-Andrade P, Cañas-Lerma AJ. Professional quality of life, engagement, and self-care in healthcare professionals in ecuador during the COVID-19 pandemic. Healthcare (Switzerland). 2021;9(5). doi: 10.3390/healthcare9050515 33946629 PMC8146458

[22] Cohen C, Pignata S, Bezak E, Tie M, Childs J. Workplace interventions to improve well-being and reduce burnout for nurses, physicians and allied healthcare professionals: a systematic review. BMJ Open. 2023 Jun 29;13(6). doi: 10.1136/bmjopen-2022-071203 37385740 PMC10314589

[23] Hevezi JA. Evaluation of a Meditation Intervention to Reduce the Effects of Stressors Associated With Compassion Fatigue Among Nurses. Journal of Holistic Nursing [Internet]. 2016 Dec 1 [cited 2023 Nov 14];34(4):343–50. Available from: https://journals-sagepub-com.ezproxy.libraries.wright.edu/doi/10.1177/0898010115615981?url_ver=Z39.88-2003&rfr_id=ori%3Arid%3Acrossref.org&rfr_dat=cr_pub++0pubmed 26598000 10.1177/0898010115615981

[24] Green AA, Kinchen V E. The Effects of Mindfulness Meditation on Stress and Burnout in Nurses. [Internet]. 2021 May 17 [cited 2023 Apr 1];39(4):356–68. Available from: https://journals.sagepub.com/doi/abs/10.1177/08980101211015818?journalCode=jhna 10.1177/08980101211015818 33998935

[25] Ramachandran HJ, Bin Mahmud MS, Rajendran P, Jiang Y, Cheng L, Wang W. Effectiveness of mindfulness-based interventions on psychological well-being, burnout and post-traumatic stress disorder among nurses: A systematic review and meta-analysis. J Clin Nurs [Internet]. 2023 Jun 1 [cited 2023 Nov 17];32(11–12):2323–38. Available from: https://pubmed-ncbi-nlm-nih-gov.ezproxy.libraries.wright.edu/35187740/10.1111/jocn.16265 35187740

[26] Lomas T, Medina JC, Ivtzan I, Rupprecht S, Eiroa-Orosa FJ. A Systematic Review and Meta-analysis of the Impact of Mindfulness-Based Interventions on the Well-Being of Healthcare Professionals. 2018 [cited 2023 Nov 17]. 10.1002/jclp.22515 28752554

[27] Armstrong JW, Tume LN. Mindfulness-based interventions to reduce stress and burnout in nurses: an integrative review. [Internet]. 2022 Feb 9 [cited 2023 Nov 17];11(1):1–11. Available from: https://www.magonlinelibrary.com/doi/10.12968/bjmh.2020.0036

[28] Scheepers RA, Emke H, Epstein RM, Lombarts KMJMH. The impact of mindfulness-based interventions on doctors’ well-being and performance: A systematic review. Med Educ. 2020 Feb 1;54(2):138–49. doi: 10.1111/medu.14020 31868262 PMC7003865

[29] Sulosaari V, Unal E, Cinar FI. The effectiveness of mindfulness-based interventions on the psychological well-being of nurses: A systematic review. Applied Nursing Research. 2022 Apr 1;64. doi: 10.1016/j.apnr.2022.151565 35307128

[30] The Science of Gratitude. 2018.

[31] Smith JA, Newman K, Marsh J, Keltner D. The gratitude project: how the science of thankfulness can rewire our brains for resilience, optimism, and the greater good. [cited 2023 Nov 16];234. Available from: https://www.newharbinger.com/9781684034635/the-gratitude-project

[32] Day G, Robert G, Rafferty AM. Gratitude in Health Care: A Meta-narrative Review. Qual Health Res [Internet]. 2020 Dec 1 [cited 2023 Nov 16];30(14):2303. Available from: /pmc/articles/PMC7649920/ doi: 10.1177/1049732320951145 32924863 PMC7649920

[33] Copeland D. Brief Workplace Interventions Addressing Burnout, Compassion Fatigue, and Teamwork: A Pilot Study. [Internet]. 2020 Jul 9 [cited 2023 Nov 16];43(2):130–7. Available from: https://journals.sagepub.com/doi/abs/10.1177/0193945920938048?journalCode=wjna 32646295 10.1177/0193945920938048

[34] Caragol JA, Johnson AR, Kwan BM. A gratitude intervention to improve clinician stress and professional satisfaction: A pilot and feasibility trial. Int J Psychiatry Med [Internet]. 2022 Mar 1 [cited 2023 Nov 16];57(2):103–16. Available from: https://pubmed.ncbi.nlm.nih.gov/33472468/ 33472468 10.1177/0091217420982112

[35] van’t Westeinde A, Patel KD. Heartfulness Meditation: A Yogic and Neuroscientific Perspective. Front Psychol. 2022;13(May). doi: 10.3389/fpsyg.2022.806131 35619781 PMC9128627

[36] Desai K, Gupta P, Parikh P, Desai A. Impact of virtual heartfulness meditation program on stress, quality of sleep, and psychological wellbeing during the covid-19 pandemic: A mixed-method study. Int J Environ Res Public Health. 2021;18(21). doi: 10.3390/ijerph182111114 34769634 PMC8583339

[37] Thimmapuram J, Pargament R, Sibliss K, Grim R, Risques R, Toorens E. Effect of heartfulness meditation on burnout, emotional wellness, and telomere length in health care professionals. J Community Hosp Intern Med Perspect [Internet]. 2017;7(1):21–7. Available from: doi: 10.1080/20009666.2016.1270806 28634520 PMC5463663

[38] Thimmapuram J, Pargament R, Bell T, Schurk H, Madhusudhan DK. Heartfulness meditation improves loneliness and sleep in physicians and advance practice providers during COVID-19 pandemic. Hosp Pract (1995) [Internet]. 2021 Aug 1 [cited 2022 Jun 17];49(3):194–202. Available from: https://pubmed-ncbi-nlm-nih-gov.ezproxy.libraries.wright.edu/33682592/33682592 10.1080/21548331.2021.1896858

[39] Mills MJ, Culbertson SS, Fullagar CJ. Conceptualizing and Measuring Engagement: An Analysis of the Utrecht Work Engagement Scale. J Happiness Stud [Internet]. 2012 Jun 7 [cited 2023 Mar 31];13(3):519–45. Available from: https://link.springer.com/article/10.1007/s10902-011-9277-3

[40] Schaufeli WB, Bakker AB, Salanova M. The Measurement of Work Engagement With a Short Questionnaire. [Internet]. 2016 Jul 2 [cited 2023 Mar 31];66(4):701–16. Available from: https://journals.sagepub.com/doi/abs/10.1177/0013164405282471?journalCode=epma

[41] A Simple Sequentially Rejective Multiple Test Procedure on JSTOR [Internet]. [cited 2023 Mar 31]. https://www.jstor.org/stable/4615733

[42] Bonamer JR, Aquino-Russell C. Self-care strategies for professional development: Transcendental meditation reduces compassion fatigue and improves resilience for nurses. J Nurses Prof Dev. 2019;35(2):93–7. doi: 10.1097/NND.0000000000000522 30741919

[43] Hevezi JA. Evaluation of a Meditation Intervention to Reduce the Effects of Stressors Associated With Compassion Fatigue Among Nurses. Journal of Holistic Nursing. 2016;34(4):343–50. doi: 10.1177/0898010115615981 26598000

[44] Asadollah F, Nikfarid L, Sabery M, Varzeshnejad M, Hashemi F. The Impact of Loving-Kindness Meditation on Compassion Fatigue of Nurses Working in the Neonatal Intensive Care Unit: A Randomized Clinical Trial Study. Holist Nurs Pract. 2023 Jul 1;37(4):215–22. doi: 10.1097/HNP.0000000000000590 37335149

[45] Thakur M, Patil Y, Philip ST, Hamdule T, Thimmapuram J, Vyas N, et al. Impact of Heartfulness meditation practice on anxiety, perceived stress, well-being, and telomere length. Front Psychol [Internet]. 2023 [cited 2023 Nov 16];14. Available from: https://pubmed-ncbi-nlm-nih-gov.ezproxy.libraries.wright.edu/37342644/ 37342644 10.3389/fpsyg.2023.1158760 PMC10278541

[46] Iyer RB, Vadlapudi S, Iyer L, Kumar V, Iyer L, Sriram P, et al. Impact of the Heartfulness program on loneliness in high schoolers: Randomized survey study. Appl Psychol Health Well Being [Internet]. 2022 [cited 2022 Sep 12]; Available from: https://pubmed-ncbi-nlm-nih-gov.ezproxy.libraries.wright.edu/35384302/ doi: 10.1111/aphw.12360 35384302 PMC10084022

[47] Thimmapuram J, Pargament R, Tredici S Del, Bell T, Yommer D, Daoud D, et al. Sleep Patterns of Resident Physicians and the Effect of Heartfulness Meditation. Ann Neurosci [Internet]. 2021 Jan 1 [cited 2023 Nov 18];28(1–2):47. Available from: /pmc/articles/PMC8558985/ doi: 10.1177/09727531211039070 34733054 PMC8558985

[48] Bartlett L, Buscot MJ, Bindoff A, Chambers R, Hassed C. Mindfulness Is Associated With Lower Stress and Higher Work Engagement in a Large Sample of MOOC Participants. Front Psychol. 2021 Sep 10;12:724126. doi: 10.3389/fpsyg.2021.724126 34566805 PMC8461060

[49] Calcagni CC, Salanova M, Llorens S, Bellosta-Batalla M, Martínez-Rubio D, Martínez Borrás R. Differential Effects of Mindfulness-Based Intervention Programs at Work on Psychological Wellbeing and Work Engagement. Front Psychol. 2021 Sep 27;12:715146. doi: 10.3389/fpsyg.2021.715146 34646205 PMC8502863

[50] Camero I, Carrico C. Addressing Nursing Personnel Burnout in Long-Term Care: Implementation of a Gratitude Journal. Holist Nurs Pract [Internet]. 2022 May 1 [cited 2023 Nov 17];36(3):E12–7. Available from: https://pubmed.ncbi.nlm.nih.gov/35435879/ doi: 10.1097/HNP.0000000000000512 35435879

[51] McGinness A, Raman M, Stallworth D, Natesan S. App-Based Three Good Things and Gratitude Journaling Incentive Program for Burnout in Pediatric Residents: A Nonrandomized Controlled Pilot. Acad Pediatr [Internet]. 2022 Nov 1 [cited 2023 Nov 17];22(8):1532–5. Available from: https://pubmed.ncbi.nlm.nih.gov/35718285/ doi: 10.1016/j.acap.2022.05.009 35718285

[52] KC A, LG RH, S M, PJ M, JB S. Gratitude at Work: Prospective Cohort Study of a Web-Based, Single-Exposure Well-Being Intervention for Health Care Workers. J Med Internet Res [Internet]. 2020 May 1 [cited 2021 Aug 27];22(5). Available from: https://pubmed-ncbi-nlm-nih-gov.ezproxy.libraries.wright.edu/32406864/10.2196/15562 PMC7256751 32406864

[53] Salvado M, Marques DL, Pires IM, Silva NM. Mindfulness-based interventions to reduce burnout in primary healthcare professionals: A systematic review and meta-analysis. Healthcare (Switzerland) [Internet]. 2021 Oct 1 [cited 2023 Nov 18];9(10). Available from: /pmc/articles/PMC8544467/ doi: 10.3390/healthcare9101342 34683022 PMC8544467

[54] Hayes SC, Levin ME, Plumb-Vilardaga J, Villatte JL, Pistorello J. Acceptance and Commitment Therapy and Contextual Behavioral Science: Examining the Progress of a Distinctive Model of Behavioral and Cognitive Therapy. Behav Ther [Internet]. 2013 Jun [cited 2023 Nov 18];44(2):180. Available from: /pmc/articles/PMC3635495/ doi: 10.1016/j.beth.2009.08.002 23611068 PMC3635495

[55] Patel KD, Pollock J (Heartfulness trainer), Soni V, Doty JR (James R. The heartfulness way: heart-based meditations for spiritual transformation. [cited 2023 Nov 18];187. https://www.newharbinger.com/9781684031368/the-heartfulness-way

[56] Seattleu S@, Velasquez M. Implementation of Mindfulness-Based Intervention to Reduce Implementation of Mindfulness-Based Intervention to Reduce Compassion Fatigue and Burnout Compassion Fatigue and Burnout IMPLEMENTATION OF A MINDFULNESS INTERVENTION 2 Executive Summary Background and Problem Statement [Internet]. https://scholarworks.seattleu.edu/dnp-projects/56

