# Supplementary material for: Impact of heartfulness meditation practice compared to the gratitude practices on wellbeing and work engagement among healthcare professionals: Randomized trial
Source: PLoS One. 2024 Jun 7;19(6):e0304093. doi: 10.1371/journal.pone.0304093 (PMC11161083; doi:10.1371/journal.pone.0304093)
Supplement: S1 File — (DOCX) [file pone.0304093.s003.docx]

On a scare of 1-10, 10 being extremely likely- how likely will you recommend

Gratitude Practices to a friend or family member? 9.18 (#11 responses)

Subject Comments

Gratitude Practices

| **Impact of GRatitude Practices on WellBeing &RElationships** |  |  |  |
| --- | --- | --- | --- |
|  |  |  |  |
| 🞏 **Increased calm**  **🞏 Puts challenges in perspective**  **🞏 Helped at home**  **🞏 Decreased stress (#3)**  **🞏 Helped control feelings**  🞏 **Able to reset**  **🞏 Increased empathy**  **🞏 Increased patience (#2)** | |  | 🞏 **Enjoyed the different gratitude practices**  **🞏 Reduced reactivity**  **🞏 Used gratitude practices more with my children than at work**  **🞏 Time management changes:**  🞏 **Taking more time for self**  **🞏 Taking more time for husband**  **🞏 Have incorporated a few gratitude practices into family life** |

| **Impact of Gratitude Practices on Professional life** |  |  | **What Would improve Gratitude practices?** |  |
| --- | --- | --- | --- | --- |
|  |  |  |  |  |
| 🞏 **Not sure if gratitude practices help**  **🞏 More accepting of work/family/boss**  **🞏 I incorporated the AWE walk**  **🞏 Helped me appreciate my own health**  **🞏 ↓stress, ↑productivity, ↑empathy, ↑focus** | |  | 🞏 **A group message board for sharing experiences**  **🞏 Podcasts were OK, but ↑visual/live communications**  **🞏 Continue podcasts sent via email** | |

| **Challenges of gratitude practices** |  |  | **Benefits of gratitude practices for the future** |  |
| --- | --- | --- | --- | --- |
|  |  |  |  |  |
| 🞏 **Not enough time in my schedule (#6)**  **🞏 Difficult to practice with all demands (#2)**  **🞏 Sometimes not in the mood to practice** | |  | 🞏 **I plan to use in difficult moments (#3)**  **🞏 Plan to re-listen to podcasts**  **🞏 Will think before making judgement**  **🞏 Gratitude practices helps me recalibrate** | |
