# Supplementary material for: Impact of heartfulness meditation practice compared to the gratitude practices on wellbeing and work engagement among healthcare professionals: Randomized trial
Source: PLoS One. 2024 Jun 7;19(6):e0304093. doi: 10.1371/journal.pone.0304093 (PMC11161083; doi:10.1371/journal.pone.0304093)
Supplement: S2 File — (DOCX) [file pone.0304093.s004.docx]

On a scare of 1-10, 10 being extremely likely- how likely will you recommend

Heartfulness Training to a friend or family member? 8.22 (#27 responses)

Subject Comments

Heartfulness Training

| **Impact Of Heartfulness Training on WellBeing & RElationships** |  |  |  |
| --- | --- | --- | --- |
|  |  |  |  |
| 🞏 **Calms/relaxes the mind (#6)**  **🞏 Improved sleep (#5)**  **🞏 Decreased stress (#3)**  **🞏 No meds for stress needed during the study**  **🞏 Improved coping**  🞏 **Felt “grounded”**  **🞏 Improved outlook**  **🞏 Increased gratitude** | |  | 🞏 **Deeper appreciation of loved ones & co-workers**  **🞏 Less reactive/calmer (#5)**  **🞏 Intentional in interactions**  **🞏 Present to the other in interactions (#4)**  🞏 **Increased empathy (#4)**  **🞏 Increased patience (#4) & less frantic (#2)**  **🞏 Heartfulness is now part of my routine** |

| **Impact of Heartfulness Training on professional life** |  |  | **What Would improve Heartfulness Training?** |  |
| --- | --- | --- | --- | --- |
|  |  |  |  |  |
| 🞏 **Improved focus on issues (#4)**  **🞏 Increased resilience/ helps me cope (#2)**  **🞏 Less fatigue (#2)**  **🞏 Decreased stress/less overwhelmed (#2)**  **🞏 Less judgemental of patients/families** | |  | 🞏 **Reduce time commitment for daily practice**  **🞏 Schedule flexibility for evening/night shift workers**  **🞏 Would like to hear about other people’s experiences with Heartfulness** | |

| **Challenges of Heartfulness Training** |  |  | **Benefits of Heartfulness Training for the future** |  |
| --- | --- | --- | --- | --- |
|  |  |  |  |  |
| 🞏 **Not enough time in my schedule (#15)**  **🞏 Difficult to practice with anxiety/racing mind**  **🞏 Need family support to practice** | |  | 🞏 **Instructor led sessions kept me accountable**  **🞏 Very helpful to manage stress, reset, create balance in life**  **🞏 Increased patience and compassion**  **🞏 Good practice for self-care** | |
