# Supplementary material for: Impact of heartfulness meditation practice compared to the gratitude practices on wellbeing and work engagement among healthcare professionals: Randomized trial
Source: PLoS One. 2024 Jun 7;19(6):e0304093. doi: 10.1371/journal.pone.0304093 (PMC11161083; doi:10.1371/journal.pone.0304093)
Supplement: S3 File — (DOCX) [file pone.0304093.s005.docx]

**Appendix A: Podcast schedule for Gratitude practice**

| **Week** | **Podcast Focus** | **Gratitude Practices Explored (#16)** |
| --- | --- | --- |
| 1. | Introduction to Gratitude Science Practices | - Awe Walk - Mental Subtraction |
| 2. | Gratitude & Self Care | - Self-Compassion Break - Self-Compassion Letter - 3 Good Things |
| 3. | Caring for One’s Thoughts | - Finding Perspective - Finding a Silver Lining - Writing a Gratitude Letter - Give it Up |
| 4. | Caring for Relationships | - Active Listening - Random Acts of Kindness - The Gift of Time - Feeling Supported |
| 5. | Gratitude & Resilience | - Goal Visualization - Give it Up. - Overcoming a Fear |

**Appendix B: Qualitative questions**

- Please describe how Heartfulness meditation or the Gratitude practice has impacted your general wellbeing (stress level, sleep quality).
- Please describe changes in your interactions with family, friends, patients, or co-workers after joining the Heartfulness meditation program or the Gratitude practice program, i.e., feeling calmer, more empathetic, or compassionate, taking more time before reacting to a situation, etc.
- Please describe if Heartfulness or the Gratitude practice has impacted your professional life in any way, i.e., better communication with patients or their families, improved focus on the assignment, less fatigue, etc.
- Would you like to continue the Heartfulness meditation practice or Gratitude practice after the program ends?
- Please describe some of the challenges or difficulties faced in incorporating the Heartfulness practice or Gratitude practice in your daily routine and what some ways you were or were not able to overcome them.
- Please suggest some ideas or feedback to improve or modify similar programs in the future.
- Please describe how you foresee the Heartfulness practice or the Gratitude practice to be beneficial to you in the future.
- How likely would you recommend the Heartfulness meditation or the Gratitude practice to a friend or family member? [Rated on a scale of 0-10; 0 - not at all likely through 10 extremely likely]

**Appendix C: Heartfulness practices**

Guided relaxation

The audio file and live sessions will have the following instructions for guided relaxation before meditation, rejuvenation, and before sleep. The audio duration will be six minutes:

1. Please sit comfortably and breathe normally. Gently close your eyes
2. Now, move your attention to your toes. Wiggle them a little and allow your toes to relax.
3. Feel very relaxing energy entering your feet from the ground, allowing your feet to relax. Let this energy slowly move up, relaxing your ankles, lower legs, calf muscles, knees, upper legs, and hips. Feel all these parts completely relaxed.
4. Allow this energy to slowly move up, relaxing your lower back and your upper back. Feel your entire back relaxed.
5. Slowly move your attention to your stomach area and allow all the muscles to relax.
6. Now, let the energy move up into your chest and let your chest deeply relax.
7. Move your attention onto your shoulders and feel as if they are melting away.
8. Let this energy slowly move into your upper arms, elbows, lower arms, hands, and fingers. Feel them completely relaxed.
9. Slowly move your attention to your neck and allow your neck muscles to relax.
10. Gently loosen your jaw and allow your chin and all the facial muscles to relax. Your lips and your eyes are relaxing. Relax your forehead.
11. Gently move into your mind and allow your mind to relax deeply and completely. Relax the top of your head.
12. Gently scan your whole body from the top of your head to the tips of your toes and feel your entire body relaxed.
13. Now, gently move into your heart and settle in there. Rest your attention on the source of light that is already present within. Do this in a very gentle and natural way.

Meditation practice

1. Participants will be asked to simply tune into their hearts and be open to any experience they may have as opposed to trying to visualize the light.
2. If their attention drifts, participants will be advised to redirect toward their hearts gently.
3. They will be asked to sit quietly for about 15-20 minutes by oneself or until the trainer says, “That is all.”
4. They will be asked to spend 3-5 minutes after meditation to observe feelings and emotions and note them.

Rejuvenation practice

1. Sit comfortably and relax with the help of audio of guided relaxation.
2. Think that you are mentally letting go of all the complexities, heaviness, and emotional burdens from your system.
3. Settle down with the thought that the complexities, heaviness, and emotional burdens are going away.
4. Think that they are going out of your whole system, through your back, from the top of your head to your tailbone.
5. Mentally suggest that they are going out of your system from your back in the form of smoke or vapor.
6. It is an active yet gentle process. Do not dwell on specific events or things you want to get rid of. Simply brush them off.
7. Gently accelerate this process with confidence and faith, and apply your will as needed.
8. If your attention drifts and you find yourself involved in other thoughts, gently bring your attention back to the rejuvenation process.
9. As the impressions are leaving from the back, you will start to feel lightness in your heart.
10. Once you feel lightness in your heart, think that a sacred current from the Source is entering into your heart from filling the vacuum left by the outgoing impressions.

Do this for about 15 minutes.
