# Supplementary material for: Impact of heartfulness meditation practice compared to the gratitude practices on wellbeing and work engagement among healthcare professionals: Randomized trial
Source: PLoS One. 2024 Jun 7;19(6):e0304093. doi: 10.1371/journal.pone.0304093 (PMC11161083; doi:10.1371/journal.pone.0304093)
Supplement: S1 Protocol — (PDF) [file pone.0304093.s006.pdf]

Impact of Heartfulness meditation practices compared to the Gratitude practice on compassion fatigue, compassion satisfaction, burnout and work engagement for healthcare professionals across United States: Randomized Trial

Background

While involved in the noble profession of helping fellow human beings, healthcare workers are exposed to a challenging work environment where they encounter distressed patients and families. Compassion and empathy are expected of all healthcare professionals though this constant demand can become overwhelming depending on the work environment, patient population, and workload. *Compassion satisfaction* is a positive, altruistic quality that describes self-appreciation while caring for and helping others. *Compassion fatigue* is the psycho-emotional distress that originates from long-term self-sacrifice coupled with prolonged exposure to difficult situations. There are two parts of compassion fatigue. The first part concerns things such as exhaustion, frustration, anger, and depression typical of burnout. *Secondary Traumatic Stress* is a negative feeling driven by fear and work-related trauma. Whereas compassion fatigue is rapid in onset, burnout is a slowly progressing disorder usually seen in a burdensome organizational environment[1].

Nurses are especially prone to reduced compassion satisfaction, higher compassion fatigue, and burnout when they experience continual physical and psychological stress originating from the care of patients, most of whom are seriously ill, and many of whom are traumatized[2]. Compassion fatigue and burnout suffered by nurses are well described in the literature. A meta-analysis of 21 studies demonstrated the prevalence rates of compassion satisfaction, compassion fatigue, and burnout were 47.55%, 52.55%, and 51.98%, respectively, among the nurses of different areas[2]. The function of a healthcare system depends on the tight integration and interdependency of physicians, nurses, and allied healthcare professions. Allied healthcare workers such as respiratory therapists, physical, occupational, and speech therapists are also expected to be exposed to similar work-related stress while caring for their patients, even though literature is lacking in this aspect[3,4]. The health administrators strive to improve the mental wellbeing of their workforce as it is essential to provide quality of care and improve patient satisfaction. Strategies such as optimal workload assignment, collegial work environment, continuing education, leadership are critical general interventions to help the healthcare workforce. Every individual tends to cope differently with the stressful work environment or challenging patient or family interactions, depending on one's inner emotional state. A healthcare administration can potentially help their healthcare workers by providing tools of self-care to better cope with day-to-day stressors associated with providing care to patients and enhance resilience.

Relaxation techniques and meditation practices have been shown to reduce compassion fatigue and burnout among healthcare providers[5–7]. These studies were limited by lack of randomization or lack of control group. The heartfulness practice comprises a morning session focusing on relaxation and meditation, an evening rejuvenation session that involves removing emotional impressions of the day, and a session at night for a deeper connection with oneself involving a

short meditation session before sleep. In addition, trainer-guided sessions are also offered. Heartfulness meditation practice has been shown to decrease loneliness & improve sleep; and reduce burnout in healthcare workers[7,8].

The American Nurses Foundation and the Greater Good Science Center (GGSC) at the University of California, Berkeley, have developed a Gratitude Practice for Nurses Toolkit to support nurses' social and emotional wellbeing. It is unclear if gratitude improves compassion fatigue and burnout and improves mental wellbeing, especially for healthcare workers, including nurses, as limited research data from randomized studies is available in this population[9.]

Work engagement is the assumed opposite of burnout. Contrary to those who suffer from burnout, engaged employees have a sense of energetic and effective connection with their work activities and they see themselves as able to deal well with the demands of their job. It would be essential to measure work engagement among healthcare workers.

This study will investigate whether heartfulness meditation practice leads to measurable changes in compassion fatigue, compassion satisfaction and burnout as well as improvement in work engagement among the healthcare providers across US compared to Gratitude practice.

The objectives of the study

The primary objective of this study is to investigate an impact of a virtual, heart-based meditation program (Heartfulness practice) guided through a Heartfulness trainer compared with a self-motivated Gratitude practice for nurses designed by the American Nurses Foundation and the Greater Good Science Center (GGSC) at the University of California, Berkeley on the burden of compassion fatigue and burnout among the nurses and allied healthcare providers.

The secondary objective is to investigate if a virtual, Heartfulness practice guided through a Heartfulness trainer results in a sustained reduction in compassion fatigue and burnout as well as improvement in work engagement among the healthcare providers.

Study Population and Recruitment Methods

We would like to implement an adaptive recruitment strategy to recruit a higher number of participants in the intervention group expecting a high attrition rate observed in previous studies where meditation practice was employed as an intervention. We would like to recruit 200 participants in the intervention group and 100 participants in the control group. Willing volunteers will be consented to, enrolled, and data will be collected and analyzed.

Inclusion and Exclusion Criteria

Inclusion criteria:

1. Adults above 18 years of age willing to participate in the study
2. Any healthcare worker involved in direct patient care at any healthcare facility within United States

3. Requires basic knowledge of the Internet and ability to follow instructions regarding email communications as well as accessing video conferences

Exclusion criteria:

1. Individuals less than 18 years of age

2. Participants with a history of meditation practice (≥ 100 hours of meditation) will be excluded from the study to avoid participant bias

Disclaimer: Any person under medical care for depression or other mental health conditions is encouraged not to participate or only participate after discussion with his/her healthcare provider so that the study participation does not interfere with current treatment.

Study design

This study will be randomized control trial where the participants will be randomly assigned to the Heartfulness meditation group (Intervention group) or the Gratitude practice group (Control group). All participants will be requested to fill out professional quality of life (ProQOL) survey and the “Participants’ Details” (including age, gender, work designation, years of experience, level of education, primary patient population etc.) forms before randomization. Participants will be subjected to computerized randomization to intervention group or Heartfulness meditation group and control group or the Gratitude practice group. All participants were requested to complete the professional quality of life (ProQOL) and , Work engagement scale at the baseline and at the end of 6-weeks, which will complete the first phase of the study.

If equal or more than 20 participants are willing to participate, we will continue with a second phase of the study where the trainer guided Heartfulness program will continue for six months to assess the long-term impact of Heartfulness practice on compassion fatigue and burnout. Email communication will be sent to all participants at the end of 6 weeks, asking for voluntary participation. Participants who volunteer for the second phase of the study will be asked to complete a same survey at three months and six months. Participants in Gratitude practice group will also be asked to complete the survey at three and six months after completion of study.

All participants in both groups will be provided with information, including recorded video presentations for Heartfulness meditation practice and the Gratitude practice, at the end of 6 weeks.

Recruitment

This study will be open to all healthcare professionals involved in direct patient care in any healthcare institution across United StatesPrincipal investigator and research team will provide information regarding the study to the general public through social media platforms such as Twitter, Facebook, and Instagram etc. with use of marketing flyers (Attached as a separate

document). We will also directly contact several healthcare institutes including Premier Health Network (Dayton, Ohio) previously familiar with Heartfulness offerings through personal and professional contacts to inform about the study.

Informed Consent

Subjects choosing to participate in the study will be sent the consent form via email, allowing them to review it. They can communicate via email where additional questions can be asked, and explanations will be provided. Secure Email communication will send and receive consent forms from subjects and limited to the study investigators. Wright State University has provided a designated secure email address:heartfulness-research@wright.edu, to be used for communication with participants. Interested participants will be sent Wright State approved survey tool; Qualtrics web link & QR code. A consent form will be attached in Qualtrics with an electronic signature function for participants to be able to provide consent for the study. We will obtain primary demographic including age, gender, work designation, years of experience, level of education, primary patient population etc. (Please see attached Participant details form) and responses to the ProQOL, Work engagement scale through Wright State University approved survey tool;Qualtrics.

Measure

The Professional Quality of Life Scale-5 (ProQOL-5) will be used to determine the risk for Compassion Fatigue (CF)[10]. ProQOL-5 uses 30 questions discretely separated into three subscales, assessing Compassion satisfaction (CS), Burn out (BO), and secondary traumatic stress (STS). The reliability of the ProQOL-5 is well documented and has established its reliability with previous research reporting a Cronbach's α score ranging from 0.71 to 0.8810. Cutoff scores of less than 43 (mild for STS/BO, severe for CS), 43 to 57 (moderate), and 57 or more (severe for STS/BO, mild for CS) were used for analysis. Cutoff scores for each participant will be calculated, and these scores will then be used to create risk profiles that show potential CF trends. Permission to use ProQOL-5 is obtained.

The Utrecht Work Engagement Scale (UWES) assesses levels of energy and mental resilience while working, along with a sense of significance, inspiration, pride, challenge, and concentration in work. Schaufeli and colleagues (2002) first proposed the UWES as a 17-item scale. Using exploratory factor analysis, they found three distinct factors of employee engagement: vigor, absorption, and dedication, consistent with their conceptualization.

All participants will be requested to provide testimonials or descriptive answers to the following qualitative questions in writing as a part of the survey at the end of 6 weeks, 3 months, and 6 months.

- Please describe how Heartfulness meditation or the Gratitude practice has impacted your general wellbeing (stress level, sleep quality)?

- Please describe changes in your interactions with family, friends, patients, or co-workers after joining the Heartfulness meditation program or the Gratitude practice program, i.e., feeling calmer, more empathetic, or compassionate, taking more time before reacting to a situation etc.

- Please describe if Heartfulness or the Gratitude practice has impacted your professional life in any way, i.e., better communication with patients or their family, improved focus on the assignment, less fatigue etc.

- Would you like to continue Heartfulness meditation practice or the Gratitude practice after the program ends?

- Please describe some of the challenges or difficulties faced to incorporate Heartfulness practice or the Gratitude practice in your daily routine and what were some ways you were or were not able to overcome them?

- Please suggest some ideas or feedback to improve or modify similar programs in future

- Please describe how you foresee Heartfulness practice or the Gratitude practice to be beneficial to you in future?

- How likely is it that you would recommend Heartfulness meditation practice or the Gratitude practice to a friend or family member? [Rated on a scale of 0-10; 0 - not at all likely through 10 extremely likely]

These data will be used for qualitative statistical analysis which will provide additional perspective to study results.

<u>Measurement of participation</u>

- Virtual sessions attendance will be recorded by investigators
- Heartfulness group: At the end of 6 weeks, participants will be asked to report frequency of home practice of meditation, rejuvenation, and relaxation before sleep (not including virtual guided sessions)
  - How many times a week have you practiced Heartfulness meditation for at least 15-20 minutes in last 6 weeks?
  - How many times a week have you practiced Heartfulness rejuvenation technique for at least 10-15 minutes in last 6 weeks?
  - How many times a week have you listened to Heartfulness relaxation audio before sleep in last 6 weeks?

- Gratitude practice group: At the end of 6 weeks, participants will be asked to report frequency of practicing Gratitude.
    - How many times a week have you practiced any techniques of the Gratitude practice in last 6 weeks?

Heartfulness meditation group

All participants in the Heartfulness Meditation group will be invited for an orientation session on the aspects of the study and the structure of the meditation protocol. Participants will also be briefed about expectations during meditation sessions and will be offered the contact details of the trainer for any further questions. There will be six additional education sessions to help the participants be comfortable with the Heartfulness practice once a week during the study period, which will be recorded and made available to the participants if they were unable to attend the live sessions. They also will receive emails with links to guided relaxation for meditation, rejuvenation, and sleep, embedded in an audio file.

- Heartfulness trainer guided virtual sessions: Guided relaxation and meditation sessions lasting 20-25 minutes will be conducted by Heartfulness trainer and primary investigator Dr. Kunal Desai every morning seven days a week. Participants will be asked to attend a minimum of two morning sessions a week.
- Guided relaxation and rejuvenation sessions lasting 15-20 minutes will be Heartfulness trainer and primary investigator Dr. Kunal Desai every morning seven days a week. Participants will be asked to attend a minimum of two evening sessions a week.
- Participants will practice meditation techniques seated in a comfortable position in their chairs during an online virtual class. They may participate in the class discussion if they choose.
- After first 6-week of study period, guided sessions will be conducted 3 times a week for the study participants willing to continue with Heartfulness practice arm for total 6 months.

Details of Virtual/Video conference calls set up and protecting privacy:

- All the sessions will be prearranged with Zoom video conferences. The details of participating in the video conference will be sent out in advance.
- A designated Zoom Enterprise account will be created. All introductory educational sessions and meditation sessions will be conducted through Zoom video conferences.
- All Zoom video sessions will be password protected. Each participant will be provided with an individual meeting ID and password to their email addresses. Participant-specific meeting ID and password will not be allowed to be shared.
- All participants' names and email addresses will be verified before allowing online video meditation or teaching sessions.
- Video sessions will NOT be recorded to maintain the privacy of the participants.

- Educational sessions will be recorded in webinar mode without inclusion of participants, to be made available for participants to review later.
- Participants will be provided with an information sheet with detailed steps to join video conferences.
- Participants will be asked to enter the initials of their first and last name with their participant ID number and will be asked to keep their videos on during virtual sessions to promote friendly interaction with a trainer.
- Participants will not be allowed to share Zoom meeting identification numbers & passwords with anyone else either privately or will be allowed to post on any social media platform (included in the participants' consent form).

Guided relaxation

The audio file and live sessions will have the following instructions for guided relaxation before meditation, rejuvenation, and before sleep. The audio duration will be six minutes:

1. Please sit comfortably and breathe normally. Gently close your eyes
2. Now, move your attention to your toes. Wiggle them a little and allow your toes to relax
3. Feel very relaxing energy entering your feet from the ground allowing your feet to relax. Let this energy slowly move up, relaxing your ankles, lower legs, calf muscles, knees, upper legs, and hips. Feel all these parts completely relaxed.
4. Allow this energy to slowly move up, relaxing your lower back and your upper back. Feel your entire back relaxed.
5. Slowly move your attention to your stomach area and allow all the muscles to relax.
6. Now, let the energy move up into your chest and let your chest deeply relax.
7. Move your attention onto your shoulders and feel as if they are melting away.
8. Let this energy slowly move into your upper arms, elbows, lower arms, hands, and fingers. Feel them completely relaxed.
9. Slowly move your attention to your neck and allow your neck muscles to relax
10. Gently loosen your jaw and allow your chin and all the facial muscles to relax. Your lips and your eyes are relaxing. Relax your forehead.
11. Gently move into your mind and allow your mind to relax deeply and completely. Relax the top of your head.
12. Gently scan your whole body from the top of your head to the tips of your toes and feel your entire body relaxed.
13. Now, gently move into your heart and settle in there. Rest your attention on the source of light that is already present within. Do this in a very gentle and natural way

Meditation practice

1. Participants will be asked to simply tune into their hearts and be open to any experience they may have as opposed to trying to visualize the light.

2. If their attention drifts, participants will be advised to redirect toward their hearts gently.
3. They will be asked to sit quietly for about 15-20 minutes by oneself or until the trainer says, "That is all."
4. They will be asked to spend 3-5 minutes after meditation to observe feelings and emotions and note them.

Rejuvenation practice

1. Sit comfortably and relax with the help of audio of guided relaxation.
2. Think that you are mentally letting go of all the complexities, heaviness, and emotional burdens from your system.
3. Settle down with the thought that the complexities, heaviness, and emotional burdens are going away.
4. Think that they are going out of your whole system, through your back, from the top of your head to your tailbone.
5. Mentally suggest that they are going out of your system from your back in the form of smoke or vapor.
6. It is an active yet gentle process. Do not dwell on specific events or things you want to get rid of. Simply brush them off.
7. Gently accelerate this process with confidence and faith, and apply your will as needed.
8. If your attention drifts, and you find yourself involved in other thoughts, gently bring your attention back to the rejuvenation process.
9. As the impressions are leaving from the back, you will start to feel lightness in your heart.
10. Once you feel lightness in your heart, think that a sacred current from the Source is entering into your heart from the filling the vacuum left by the outgoing impressions.
11. Do this for about 15 minutes.

Home practice:

- In the morning, the participants will be asked to listen to an audio file consisting of the Heartfulness relaxation technique followed by meditation for about 15-30 minutes in place of or in addition to guided sessions as per the convenience of the participants.
- In the evening or at the end of the day's work, the participants will be asked to listen to an audio file consisting of the Heartfulness relaxation technique followed by rejuvenation for about 15-20 minutes in place of or in addition to guided sessions as per the convenience of the participants.
- At night, participants were asked to listen to an audio file consisting of the Heartfulness relaxation technique for sleep. This was to be done in a supine position, and the instructions were similar to the morning meditation technique. This practice was suggested to be followed on a daily basis.

- Participants will be asked to self-report the adherence to the intervention at the end of the 6-week study period.

Certified Heartfulness Trainers

Dr. Kunal Desai (PI) is a physician and a certified Heartfulness trainer who will provide education, training and conduct all virtual meditation sessions during this study.

If any unforeseen situation forces an absence of Dr. Kunal Desai, Co-Primary investigator: Dr. Alpa Desai will start a Video meeting (without video interaction between participants and trainer) and invite one of the alternate Heartfulness trainers (Shanthi Venkat, Prasad Venkat, Dr Chandra Koneru) to maintain the privacy of the participants.

Gratitude practice group

All participants in the Gratitude practice group will be invited for an orientation session on the aspects of the study and review of the toolkit for cultivating the practice of gratitude in nursing. This program created by the American Nurses Foundation and the Greater Good Science Center (GGSC) at the University of California, Berkeley, is designed to support nurses' social and emotional wellbeing. This toolkit is based on scientific research revealing the many benefits of gratitude. This toolkit provides different ways for individuals to express gratitude during their daily interactions and work environment, such as writing a gratitude letter, taking a savoring walk, a few minutes before and after each patient encounter, during staff huddle, etc. Participants would have an opportunity to participate in a 21-days gratitude challenge by registering on www.Thnx.org if offered during the study period. All participants will be sent an email once a week during the study period to provide a gentle reminder to practice gratitude. Participants will be asked to attend an orientation session via Zoom about Gratitude Science for 1 hour. If the participant cannot attend the meeting, they can review the recorded video for information. Participants will be provided with information to access podcast each week on gratitude practice during the 6-week study period. Tool kit available at: https://ggsc.berkeley.edu/gratitudefornurses?_ga=2.59198189.1166833940.1621437217-1764624032.1620078854. Subjects in the control group will also complete the ProQOL-5 at the beginning and end of the six weeks and after 3 & 6 months unless they start practicing Heartfulness meditation.

Data to be utilized

Participant Information from the Participant details form (attached)

Data from standardized assessment tools

From the Professional Quality of Life scale (attached)

From the Utrecht Work Engagement Scale (UWES)

Data will be extracted from the information collected from enrollment, assessment and classes and will be stored in a secured file only accessed by the PI.

Data analysis will include descriptive statistics, regression, and repeated measures ANOVA to assess the impact of any intervention on work engagement, compassion fatigue and burnout.

Risks and Risk Management

For Heartfulness Meditation: Some participants may have limited endurance and decreased strength related to their health conditions. Some may also have concurrent medical problems such as arthritis or other challenges. Participants receive written and verbal instructions to never do anything that they feel is dangerous or painful to them.

In the practice of meditation, although calmness is induced, there can also be the experience of unwanted emotions and thoughts arising and passing. There may also be bodily discomfort through sitting for a prolonged period. If the participant is unable to continue meditation due to this, they may open their eyes briefly, take a slow deep breath, and then close their eyes to continue to meditate, or alternatively, they may leave if that is their desire.

The recognition of this is part of the long-term benefit of the course, but it can be upsetting or uncomfortable in the short term. To address this, participants may change their posture to continue meditation. Participants have their first experience of the technique in the supportive atmosphere of the virtual classroom and are taught what to expect and how to cope with experience as it unfolds. They also have contact information from the trainer, and participants may call or email the trainer with any questions or concerns. If any experience in class reveals intent to self-harm, the investigators will follow up promptly, and participants will be advised to contact emergency room or primary care physician.

For the Gratitude Science education, there is minimal risk. The education program is freely available on the web.

As for both interventions, Participants often share their experiences in the group setting; therefore, confidentiality applies to trainers, educators, and group members. At the first Heartfulness Medication class, participants sign an agreement that they will hold class events confidentially and not give each other advice.

Benefits to Subjects

Participants who complete the course either in the Heartfulness meditation group or the Gratitude practice group may experience reduced compassion fatigue and burnout and may have an improved sense of emotional wellness and improvement in the quality of sleep.

Compensation/Incentives and Research-related Costs

There will be no compensation, incentive, or research-related costs to the participants associated with the study.

Alternatives/Choice to Participants

Subjects may choose not to participate in this project with no effect on their current situation. Participants may also choose to withdraw from the program at any time with no consequences. There is no fee/charge for either program.

Research Materials, Records and Confidentiality

All information will be drawn from the assessment tools previously described. Medical records will not be utilized in this project. Data will be reported in aggregate so that no individual health information is revealed.

Identifiable information will be gathered initially then replaced by a survey identification number to protect each participant's identity. Other assessment forms will be identifiable by survey identification number.

All collected data will be stored on a secure password-protected file on Wright State University provided secured Proofpoint Secure Share.

Intended Use of Research

The investigators plan to present study results in a poster format at conferences related to medical research and evidence-based practice and present to any relevant medical committees. The investigators will disseminate the findings at professional conferences and through publication. One or both programs may demonstrate a benefit for healthcare organizations.

Required Resources

Heartfulness Institute provides all the guided relaxation and meditation sessions through volunteer Heartfulness trainers free of charge throughout the globe. Other than a subscription for the Zoom Enterprise account, no other investment or expenses are expected. The Gratitude Science Education and written materials are also free. All participants are volunteers. It applies without any exceptions for the study of this purpose as well.

1. Slatten LA, David Carson K, Carson PP. Compassion fatigue and burnout: What managers should

know. *Health Care Manag (Frederick)*. 2011;30(4):325-333. doi:10.1097/HCM.0b013e31823511f7

2. Zhang YY, Han WL, Qin W, et al. Extent of compassion satisfaction, compassion fatigue and burnout in nursing: A meta-analysis. *J Nurs Manag*. 2018;26(7):810-819. doi:10.1111/jonm.12589

3. Sorenson C, Bolick B, Wright K, Hamilton R. Understanding Compassion Fatigue in Healthcare Providers: A Review of Current Literature. *J Nurs Scholarsh*. 2016;48(5):456-465. doi:10.1111/jnu.12229

4. Smart D, English A, James J, et al. Compassion fatigue and satisfaction: A cross-sectional survey among US healthcare workers. *Nurs Heal Sci*. 2014;16(1):3-10. doi:10.1111/nhs.12068

5. Hevezi JA. Evaluation of a Meditation Intervention to Reduce the Effects of Stressors Associated With Compassion Fatigue Among Nurses. *J Holist Nurs*. 2016;34(4):343-350. doi:10.1177/0898010115615981

6. Bonamer JR, Aquino-Russell C. Self-care strategies for professional development: Transcendental meditation reduces compassion fatigue and improves resilience for nurses. *J Nurses Prof Dev*. 2019;35(2):93-97. doi:10.1097/NND.0000000000000522

7. Thimmapuram J, Pargament R, Sibliss K, Grim R, Risques R, Toorens E. Effect of heartfulness meditation on burnout, emotional wellness, and telomere length in health care professionals. *J Community Hosp Intern Med Perspect*. 2017;7(1):21-27. doi:10.1080/20009666.2016.1270806

8. Thimmapuram J, Pargament R, Bell T, Schurk H, Madhusudhan DK. Heartfulness meditation improves loneliness and sleep in physicians and advance practice providers during COVID-19 pandemic. *Hosp Pract (1995)*. Published online March 8, 2021. doi:10.1080/21548331.2021.1896858

9. KC A, LG R-H, S M, PJ M, JB S. Gratitude at Work: Prospective Cohort Study of a Web-Based, Single-Exposure Well-Being Intervention for Health Care Workers. *J Med Internet Res*. 2020;22(5). doi:10.2196/15562

10. H U D N A B, Stamm LL, Maynard B, Bercier M. The Concise ProQOL Manual Related papers T he Secondary Effect s of Helping Ot hers: A Comprehensive Bet h H U D N A L L St amm Int ervent ions for Secondary Traumat ic St ress wit h Ment al Healt h Workers: A Systemat ic Review.

11. Pisanti, R., Paplomatas, A., & Bertini, M. (2008). Measuring the positive dimensions among health care workers: a contribution to the Italian validation of the UWES--Utrecht Work Engagement Scale. *Giornale Italiano di Medicina del Lavoro ed Ergonomia*, *30*(1 Suppl A), A111-9..
